# Reinforcing Particle Architectural Stability of Li‐Rich Cathode With Enhanced Anionic Redox Reaction Reversibility

**DOI:** 10.1002/anie.6490997

**Published:** 2026-08-14

**Authors:** Yizhen Huang, Bixian Ying, Chunpu Li, Maolin Yang, Kang Zhang, Guifan Zeng, Jiyuan Xue, Yuan Tian, Wei Li, Chunjing Hu, Tao Zeng, Peng Zhang, Chao Li, Yinguo Xiao, Huaqiang Chen, Tian Qiu, Stefan Schuppler, Peter Nagel, Jin Yi, Changming Qu, Jia‐Wei Wang, Fanghua Ning, Qingsong Wang, Yu Qiao, Shi‐Gang Sun

**Affiliations:** ^1^ State Key Laboratory of Physical Chemistry of Solid Surfaces Department of Chemistry College of Chemistry and Chemical Engineering Xiamen University Xiamen P. R. China; ^2^ Battery Research center (MEET) University of Muenster Muenster Germany; ^3^ School of Physics East China Normal University Shanghai P. R. China; ^4^ School of Advanced Materials Peking University Shenzhen Graduate School Shenzhen China; ^5^ Huayou New Energy Technology (Quzhou) Co., Ltd Quzhou Zhejiang P. R. China; ^6^ Institute For Quantum Materials and Technologies Karlsruhe Institute of Technology Karlsruhe Germany; ^7^ Karlsruhe Nano Micro Facility Karlsruhe Institute of Technology Eggenstein‐Leopoldshafen Germany; ^8^ Institute For Sustainable Energy College of Sciences Shanghai University Shanghai China; ^9^ Amperex Technology Limited (ATL) Ningde P.R. China; ^10^ College of New Energy and Materials Ningde Normal University Ningde P.R. China; ^11^ Bavarian Center for Battery Technology Department of Chemistry University of Bayreuth Bayreuth Germany

**Keywords:** anionic redox reaction reversibility, internal compactness, lithium‐rich cathode, particle architectural stability

## Abstract

Lithium‐rich layered oxides (LRLOs) promise exceptional energy densities (∼1000 Wh kg^−1^) but suffer from coupled structural and chemical instabilities that impede their commercialization. While atomic scale regulation of anionic redox reaction (ARR) reversibility has been extensively explored, the mesoscale architectural degradation, which is exacerbated by detrimental porous structures inherited from precursors, remains critical yet overlooked. Herein, a cross‐scale synergistic strategy was introduced to simultaneously reinforce the mesoscale particle architecture and stabilize the atomic‐scale charge compensation in LRLOs. By incorporating fluorine to modulate the local electronic environment, the surface energy of the active (010) facets is selectively lowered, driving a profound morphological transformation of primary particles from plate‐like to equiaxed. This thermodynamic reconstruction effectively disrupts the adverse precursor‐inherited porosity, yielding highly compact secondary particles with accelerated Li^+^ diffusion kinetics. Simultaneously, at the atomic scale, the strengthened transition metal‐oxygen (TM‐O) covalency suppresses excessive oxygen activation, thereby enhancing reversibility of the ARR. Benefiting from this synergistic architectural and chemical modulation, the cathode exhibits a 6.8% increase in Coulombic efficiency and delivers exceptional long‐term stability with capacity retention of 88.8% after 950 cycles. For Ah‐level full cells, 96.3% of reversible capacity after 1500 cycles under high‐voltage is achieved.

## Graphical abstract

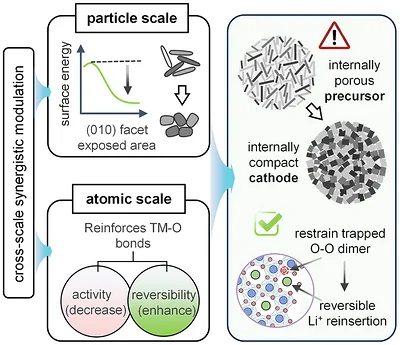

## 1 Introduction

Layered oxide cathode materials have emerged as a primary focus of research in the lithium‐ion battery (LIB) field, owing to their remarkably high energy density, environmental sustainability, and improved safety and reliability. Among these materials, lithium‐rich layered oxides (LRLOs) deliver a specific capacity approaching the theoretical limit through synergistic redox reactions involving transition metals (TM) and lattice oxygen [1, 2, 3, 4]. Their performance significantly exceeds that of conventional layered oxide cathodes, positioning them as one of the most promising candidates for next‐generation high‐energy‐density LIBs [5, 6]. However, two fundamental structural challenges severely impede their widespread commercialization: (i) the low particle architectural stability stemming from the porous structure of precursors, and (ii) the inherent irreversibility of the anionic redox reaction (ARR), which triggers structural degradation and voltage decay [7, 8, 9]. Therefore, the multi‐scale challenges of LRLOs, spanning from the mesoscale particle to the atomic‐scale local environment, must be systematically addressed to enable their large‐scale application.

At the mesoscale, the primary particles of most LRLOs and commercial nickel‐cobalt‐manganese layered oxides (NCM) share a similar plate‐like morphology with their precursors. However, their stacking modes differ fundamentally, and this difference directly governs the internal compactness of the corresponding secondary particles. Specifically, NCM are often processed via a precise co‐precipitation technique, through which a radial alignment and compact stacking of primary particles are achieved within secondary particles, enabling a dense and mechanically robust internal structure [10, 11, 12, 13]. In contrast, due to the high manganese content in LRLOs, their primary particles tend to exhibit random layout and lose compact packing. This precursor‐inherited porosity intrinsically hinders the formation of highly compact secondary particles, reducing the volumetric energy density and the mechanical integrity of the final cathode [14, 15]. Under continuous cycling, these porous particles are highly susceptible to crack propagation and severe fracturing. This creates freshly exposed active surfaces, which not only disrupt the internal conductive network but also accelerate electrolyte permeation and continuous interfacial side reactions, ultimately leading to rapid capacity decay.

At the atomic scale, the irreversibility of ARR constitutes another critical bottleneck for LRLO cathodes. The ARR primarily originates from Li─O─X local coordination environments in Li_2_MnO_3_‐like domains, where oxygen anions are weakly bonded and electrochemically active [16]. Upon high‐voltage charging (> 4.5 V vs. Li^+^/Li), charge compensation involves lattice oxygen oxidation, generating both oxygen loss (via O_2_ release) and oxidized oxygen species retained within the lattice, such as oxygen holes and O─O dimers. While TM redox (e.g., Ni) is largely reversible and oxygen release can be partially compensated by Mn reduction [17], the overall capacity reversibility is predominantly dictated by the reversibility of lattice oxygen. In particular, irreversible species (e.g., trapped oxygen dimers) remain immobilized within the lattice, thereby blocking Li^+^ re‐intercalation, preventing associated Mn redox compensation, and causing persistent capacity loss. Moreover, these oxygen species drive TM migration and layered‐to‐spinel/rock‐salt transformation, accelerating structural degradation and voltage decay [7, 18]. Crucially, this atomic‐scale instability is exacerbated by the mesoscale porous architecture, which facilitates oxygen accumulation and unconstrained release, thereby elevating safety risks in practical pouch cells.

Despite these interconnected degradation mechanisms, current efforts have predominantly focused on atomic‐scale structural regulation, which is unfortunately insufficient to enable the commercialization of LRLO. Electrochemical performance is governed by the integrated effects of cross‐scale structural characteristics. Therefore, a collaborative design strategy is urgently needed to simultaneously regulate the local covalent environment and mesoscale particle morphology. It should also remain compatible with manufacturing cost and processing constraints. However, such cross‐scale synergistic optimization remains largely unexplored.

In this work, to achieve the reinforced mesoscale architectural stability and enhanced reversibility of atomic scale ARR, LRLO materials were systematically optimized by incorporating a precise amount of fluorine. At the mesoscale, regulation of (010) facet exposure has been extensively investigated in conventional layered oxide cathodes, primarily to enhance Li^+^ diffusion kinetics. However, its role in LRLO systems requires a fundamental redefinition of design principles due to the intrinsically porousness of their secondary particle architecture. In this context, the primary particle morphology of LRLO was engineered through the regulation of crystal‐plane surface energy, driving a transition from plate‐like to equiaxed shapes. This transformation significantly increased the internal compactness of the secondary particles. Simultaneously, at the atomic scale, an O‐Mn‐F coordination framework was constructed to modulate the local charge distribution and the TM‐oxygen (TM‐O) bonding environment. This modulation suppressed excessive oxygen activation while enhancing ARR reversibility. Through this cross‐scale synergistic regulation strategy, a clear and feasible material design path was established. This balanced optimization framework, integrating fundamental innovation with processing feasibility, provides coordinated guidance for both academic research and industrial development, facilitating the practical implementation of high‐capacity LRLO cathodes.

## 2 Results and Discussion

### 2.1 Internal Compactness and Its Microstructural Inheritance From Precursor to Cathodes With Different Ni/Mn Ratios

To compare the effects of different Ni/Mn ratios on internal morphology, a comprehensive cross‐sectional analysis of secondary particles was conducted. Six commercially available precursors and their corresponding derived cathode materials were characterized using focused ion beam‐scanning electron microscopy (FIB‐SEM). The precursors investigated include: Ni_0.9_Mn_0.05_Co_0.05_(OH)_2_ (Ni90), Ni_0.8_Mn_0.1_Co_0.1_(OH)_2_ (Ni80), Ni_0.6_Mn_0.2_Co_0.2_(OH)_2_ (Ni60), Ni_0.5_Mn_0.50_(OH)_2_ (Ni50), Ni_0.40_Mn_0.60_(OH)_2_ (Ni40), and Ni_0.35_Mn_0.65_(OH)_2_ (Ni35). The corresponding derived cathode materials are: LiNi_0.9_Mn_0.05_Co_0.05_O_2_ (Ni90, NCM), LiNi_0.8_Mn_0.1_Co_0.1_O_2_ (Ni80, NCM), LiNi_0.6_Mn_0.2_Co_0.2_O_2_ (Ni60, NCM), Li_1.08_Ni_0.46_Mn_0.46_O_2_ (Ni50, LRLO), Li_1.09_Ni_0.36_Mn_0.55_O_2_ (Ni40, LRLO), and Li_1.13_Ni_0.30_Mn_0.57_O_2_ (Ni35, LRLO), respectively. As shown in Figure 1a, the internal compactness of the secondary particles progressively decreases as the Ni/Mn ratio in the precursor is reduced. Specifically, when the Ni content falls to the critical threshold of 50%, the stacking behavior of the primary particles undergoes a transition to a porous internal structure. This porous morphology becomes even more pronounced when the Ni content is further reduced to 35%. Therefore, while the design of low‐nickel precursors aligns with current development trends, the emergence of this porous architecture introduces a significant structural defect. Critically, consistent with the well‐established “hereditary” characteristics of precursor morphology, this porous internal structure is inevitably transferred to the derived cathode after high‐temperature sintering (Figure 1b). High‐nickel precursors are typically used to synthesize NCM cathode. In these cathodes, the primary particles exhibit a plate‐like morphology with a radially aligned, compact arrangement, resulting in low porosity. In contrast, low‐nickel precursors are commonly used to prepare LRLO cathodes, which preserve a morphology similar to that of the original precursors. The primary particles display a plate‐like morphology with a random, loose arrangement, leading to a highly internal porous structure. Although the internal porosity of cathodes derived from hydroxide precursors is significantly lower than that of those derived from carbonate precursors, it remains considerably higher than that observed in NCM cathodes [14]. This porous structure of LRLO has become an inherent defect, reducing the architectural stability of the particles, which leads to low volumetric energy density, poor compressive strength, and more severe interfacial degradation. These issues represent the main bottlenecks hindering the commercial viability of LRLO. To enhance the internal compactness of secondary particles, precise control over the exposure ratio of specific crystal facets in primary particles during high‐temperature sintering was introduced while retaining the advantages of the low‐Ni/high‐Mn composition design. Consequently, the volumetric energy density, as well as the mechanical and interfacial stability of the LRLO cathode material, were enhanced (Figure 1c).

**FIGURE 1:**
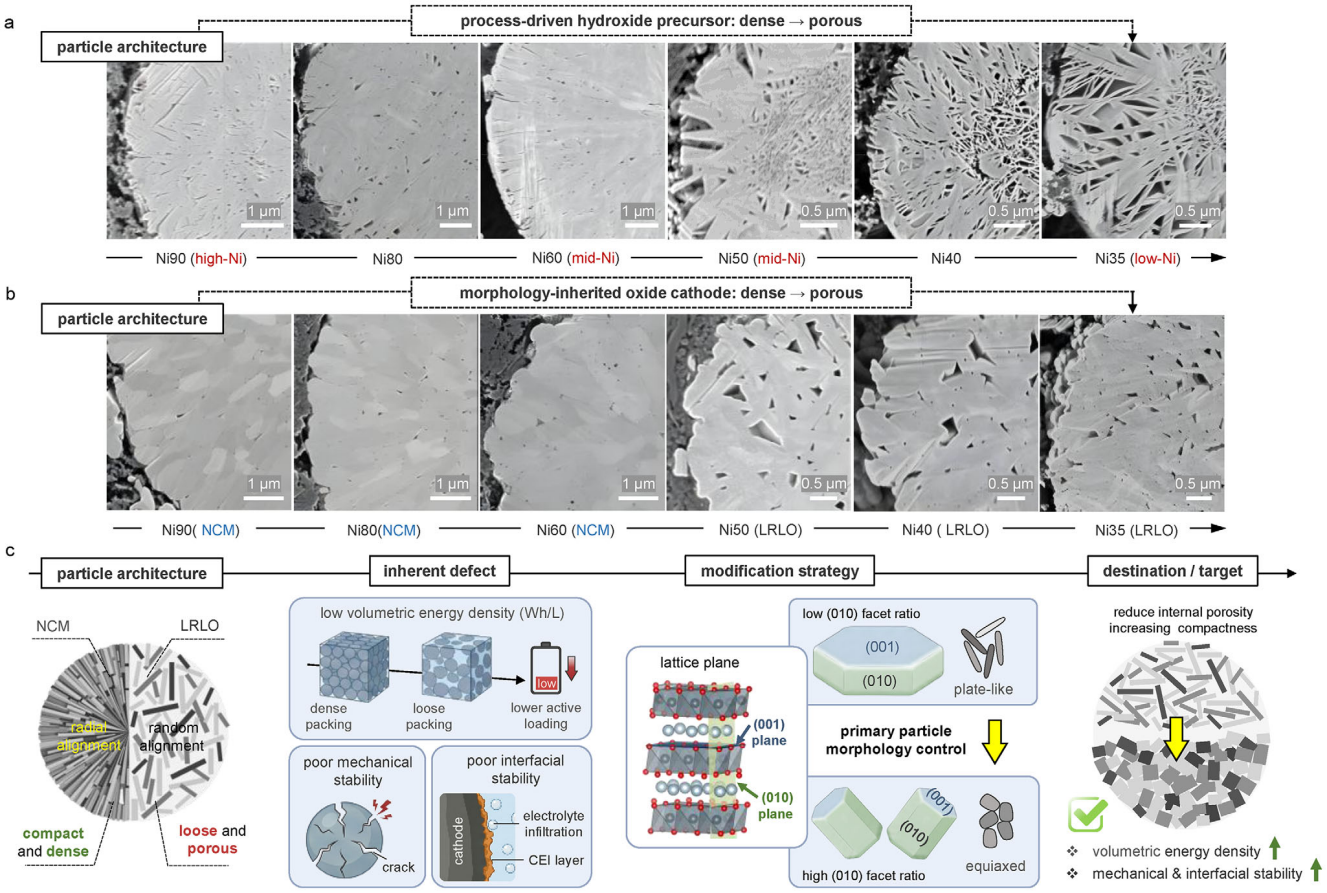
Particle architecture evolution in cathodes with varying nickel content. Cross‐sectional SEM images of (a) the precursor and (b) the corresponding cathodes derived, ranging from high‐nickel (Ni90) to low‐nickel (Ni35). (c) Schematic illustrating the relationship between particle architecture, inherent defects, modification strategies, and the final cathode materials.

### 2.2 Thermodynamic Facet Modulation for Morphological Reconstruction

According to the Gibbs‐Wulff theorem [19, 20], the equilibrium morphology of a crystal is fundamentally determined by the minimization of its total surface free energy for a given volume. Therefore, crystal facets with higher surface energy tend to exhibit smaller exposed areas and may ultimately disappear from the equilibrium morphology. In hexagonal layered oxide cathode material (e.g., NCM, LRLO), crystal growth is typically highly anisotropic: high‐energy planes (e.g., (010)) progressively diminish during synthesis, while thermodynamically stable low‐energy planes (e.g., (001)) become dominant. This surface energy‐driven growth restricts extension along the *c*‐axis and favors rapid growth within the *ab*‐plane, typically producing plate‐like‐shaped primary particles with (001) planes as the predominant exposed facets [21, 22, 23]. However, such anisotropic primary particle morphologies often hinder dense packing during the formation of secondary particles, leading to a series of adverse effects. It has been reported that precise modulation of the surface energy of specific crystal planes can effectively control their relative exposure of these planes during crystal growth, thereby enabling the directional design of primary particle morphology [24, 25]. Guided by this principle, we strategically utilized highly electronegative fluorine to modulate local electronic structures and alter surface energies. We expect that introducing a fluorine source with a content of 1 mol% during solid‐state synthesis (sample denoted as LR‐F, with a fluorine‐free control sample Li_1.13_Ni_0.30_Mn_0.57_O_2_ denoted as LR) reduces the surface energy of the (010) planes. This disrupts the preferential exposure of the (001) facets and promotes the exposure of (010) facets, driving the morphological transformation of the primary particle morphology from plate‐like to equiaxed, thereby ultimately enhancing the compactness of the secondary particles.

Such variations in primary particle morphology and crystallographic facet exposure (preferred orientation) can be indirectly probed by x‐ray diffraction (XRD). Specifically, within the *R* $\bar{3}$ *m* space group, the physical (001) facet is parallel to the crystallographic (003) diffraction planes. During powder XRD measurements, the preferred (003) plane leads to an increased X‐ray scattering probability, resulting in enhanced diffraction intensity. Consequently, the *I*_(003)_/*I*_(104)_ intensity ratio serves as a reliable semi‐quantitative descriptor to evaluate the morphological anisotropy and the relative exposure of the (001) facets. Ex situ synchrotron XRD (sXRD) was employed to systematically track the structural evolution during the solid‐state reaction (300 °C–900 °C). As shown in Figure 2a,b, while the phase evolution pathways of LR and LR‐F are comparable, pronounced differences emerge in their *I*_(003)_/*I*_(104)_ ratios. A higher *I*_(003)_/*I*_(104)_ ratio for the LR cathode indicates severe preferred orientation induced by enhanced exposure of the (001) facet, corresponding to plate‐like primary particles. In contrast, the significantly lower *I*_(003)_/*I*_(104)_ ratio in the LR‐F sample indicates suppressed preferred orientation, which is a direct consequence of reduced (001) facet and increased (010) facet exposure, perfectly aligning with the formation of randomly packed equiaxed primary particles (Figure 2c).

**FIGURE 2:**
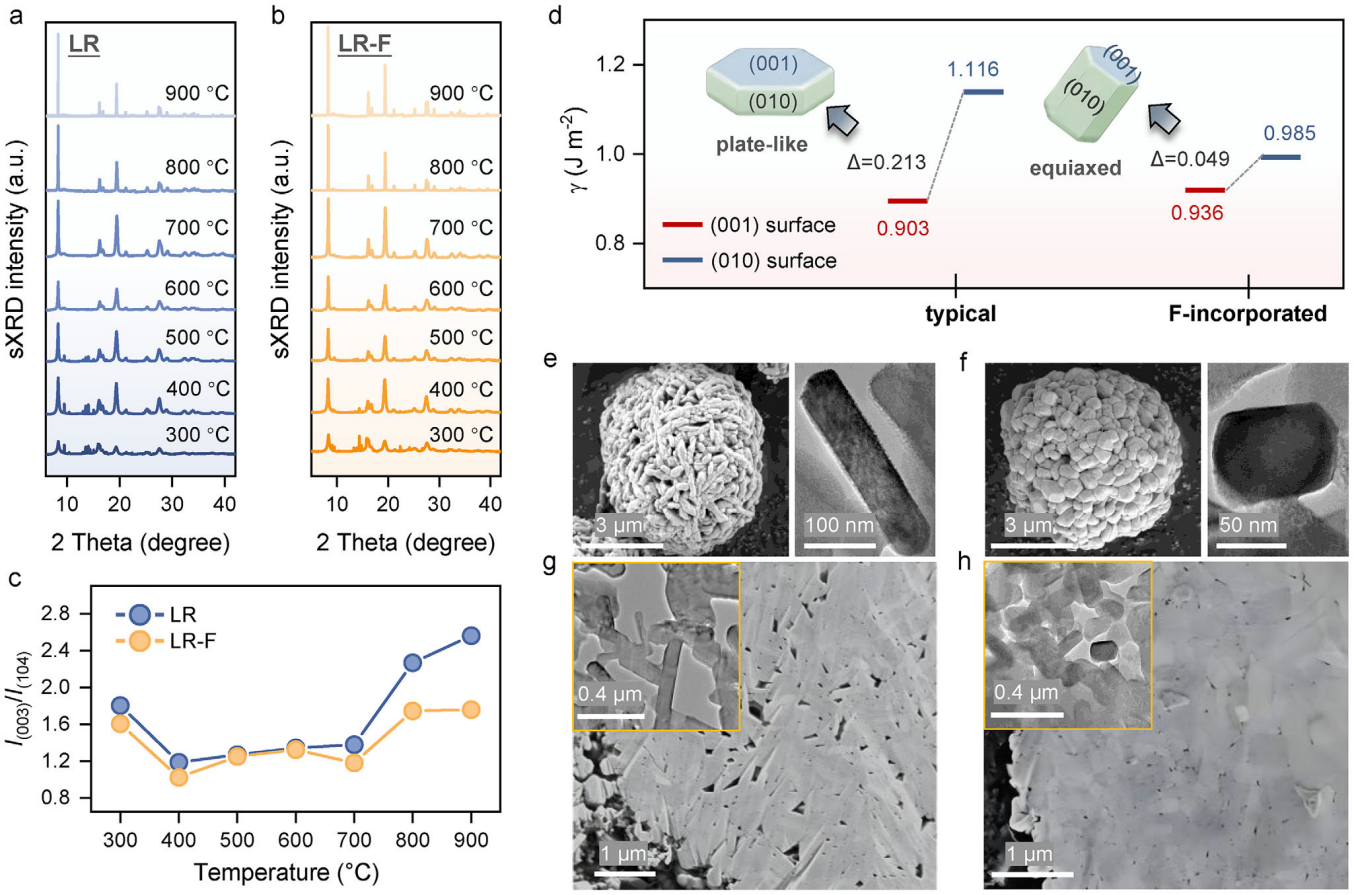
Preferential crystal facet growth and internal structural densification. Ex situ synchrotron x‐ray diffraction (sXRD) of (a) LR and (b) LR‐F cathode. (c) sXRD‐measured *I_(003)_*/*I_(104)_* ratio as a function of synthesis temperature. (d) Theoretical calculation results for adsorption energy and surface energy. SEM (left) and TEM (right) images of (e) LR and (f) LR‐F cathodes. Cross‐sectional morphology of (g) LR and (h) LR‐F cathodes. Insert: Cross‐sectional TEM image showing the detailed morphology of the region of interest.

To elucidate the thermodynamic origin of this transformation, density functional theory (DFT) calculations were performed, with the corresponding surface structure models provided in Figures S1 and S2. As shown in Figure 2d, fluorine incorporation significantly reduces the surface energy disparity between the high‐energy (010) and low‐energy (001) facets in the *R* $\bar{3}$ *m* layered structure, decreasing from 0.213 J m^−2^ to 0.049 J m^−2^. This significantly narrowed energy gap suppresses the preferential growth of the (001) facets and leads to more comparable growth rates between the two crystallographic planes, thereby inhibiting anisotropic crystal growth. Consequently, the particle morphology evolves from plate‐like to equiaxed structures, accompanied by an increased exposure of (010) facets. To experimentally validate this prediction, FIB‐SEM and transmission electron microscopy (TEM) were employed to characterize the surface and cross‐section morphologies of the secondary particles. The two samples exhibit similar spherical secondary particles with diameters of ∼6 µm, ensuring a fair structural comparison. Notably, their primary particle morphologies differ significantly: LR shows plate‐like‐shaped primary particles typical of conventional LRLO cathodes (Figures 2e and S3a), whereas LR‐F displays equiaxed ones (Figures 2f and S3b), as further confirmed by high‐magnification TEM images. Cross‐sectional analysis (Figure 2 g,h) reveals that LR possesses a porous and loosely packed interior, while LR‐F exhibits a markedly more compact architecture with reduced porosity, consistent with TEM observations. Furthermore, a series of control samples with varying fluorine contents were synthesized to systematically investigate the relationships among fluorine content, particle morphology, and internal compactness. Specifically, samples with fluorine contents of 0.5 mol% and 2 mol% were prepared and denoted as LR‐F‐0.5 and LR‐F‐2, respectively. As the fluorine content increases, the aspect ratio of the primary particles continuously decreases, accompanied by a gradual transition from plate‐like to equiaxed morphology. Simultaneously, the internal compactness of the secondary particles increases monotonically (Figure S4). These results suggest that fluorine incorporation induces a morphological transition of the primary particles, facilitating denser particle packing and thereby enhancing the internal compactness of the secondary particles.

To further quantify the evolution of the exposed (010) facets, full‐pattern Rietveld refinement was performed using the March–Dollase preferred‐orientation model (Figure S5 and Table S1). By defining the (010) direction as the preferred‐orientation axis, this model enables quantitative evaluation of the morphology‐induced preferred orientation while maintaining the intrinsic structural parameters during refinement. The refined March parameter(*r*) decreases from 1.104 for the pristine LR sample to 0.861 for the LR‐F sample. According to the March–Dollase correction function (as detailed in the Experimental Procedures of the Supporting Information), the corresponding orientation factor (*P*) for the (010) reflection increases from 0.74 to 1.57, indicating a pronounced enhancement of the preferred orientation along the (010) direction. This result is consistent with the SEM observations, which reveal the morphological evolution of primary particles from elongated rod‐like crystallites to a more equiaxed morphology. Collectively, these results quantitatively demonstrate that fluorine incorporation modifies the crystallite habit and preferred orientation, resulting in an increased relative exposure of the electrochemically active (010) facets.

Collectively, these results corroborate the DFT prediction that the fluorine incorporation effectively drives the transformation from plate‐like to equiaxed primary particle morphology, thereby modifying their packing behavior and ultimately enhancing the internal compactness of the secondary particles [26].

Such variations in primary particle morphology and crystallographic facet exposure are intrinsically linked to Li^+^ transport kinetics. In the layered structure with *R* $\bar{3}$ *m* symmetry, Li^+^ diffusion is strictly confined to the 2D pathways within the *ab*‐plane. The (001) basal plane is oriented parallel to the close‐packed transition TM‐O slabs. Because these slabs block ionic movement along the *c*‐axis, the wide (001) surfaces typical of anisotropic particles are fundamentally electrochemically inactive for Li^+^ insertion/extraction. In contrast, the (010) facets (and symmetrically equivalent planes) are parallel to the *c*‐axis, directly exposing the edges of the lithium layers and providing accessible entrances for the 2D Li^+^ diffusion channels. Consequently, the exposed area fraction of the (010) facets act as a critical structural descriptor, governing the number of active sites available for interfacial Li^+^ flux.

Specifically, the thermodynamic transformation of primary particles from plate‐like to equiaxed plays a dual role in optimizing diffusion kinetics (Figure 3a). First, the shift to an equiaxed geometry significantly reduces the lateral dimension of the basal plane, thereby shortening the intra‐particle solid‐state diffusion length for Li^+^ migration from the bulk to the surface. Second, it substantially increases the proportion of electrochemically active (010) facets, maximizing the availability of surface‐accessible diffusion pathways. As a result of this simultaneous reduction in transport distance and expansion of active interface, the overall Li^+^ diffusion is remarkably accelerated. This fundamental enhancement in Li^+^ transport kinetics directly translates into the superior rate capability of the engineered electrode. To verify this, rate performance measurements were conducted to evaluate the kinetic behavior of the LR and LR‐F cathodes. As shown in Figure 3b, at low current densities (0.1C, 1C = 200 mA h g^−1^), where kinetic limitations are negligible and capacity is primarily governed by the availability of active sites, the LR‐F cathode exhibits a higher discharge capacity (285.3 mAh g^−1^) than the LR counterpart (260.1 mAh g^−1^), suggesting that F incorporation helps preserve a greater number of electrochemically active Li storage sites. As the current density increases and kinetic limitations become more pronounced, the superiority of the LR‐F cathode becomes more evident. At 5C, it delivers a discharge capacity of 186.1 mA h g^−1^, exceeding that of the LR cathode by 76.1 mA h g^−1^. This result confirms that the increased exposure of (010) facets effectively reduce Li^+^ diffusion resistance and enhances transport kinetics, which is further supported by the corresponding discharge curves (Figure S6) at various rates.

**FIGURE 3:**
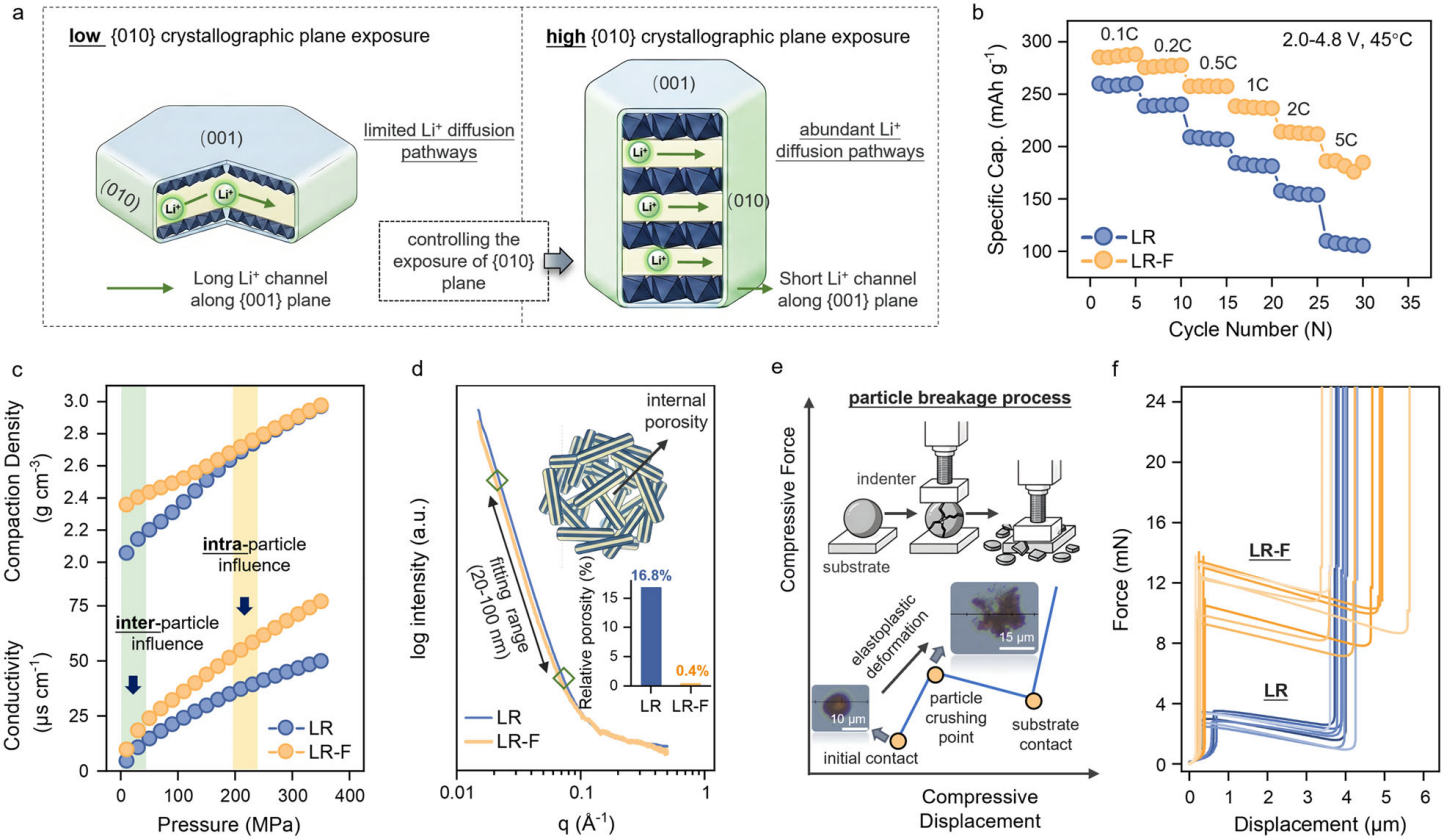
Effects of variations in the exposed area ratio of specific crystal facets. (a) Schematic illustration of Li^+^ diffusion pathways under conditions of low and high (010) facet exposure. (b) Rate performance at different rates of LR and LR‐F cathodes at 45 °C, 2.0–4.8 V. (c) Compaction density and electronic conductivity of LR and LR‐F. (d) SAXS profiles and corresponding internal porosity statistics of LR and LR‐F. Inset: The corresponding fitting results. (e) Single particle compressive crushing behavior and (f) its corresponding pressure‐displacement curve.

To further elucidate the Li^+^ transport kinetics, Cyclic voltammetry (CV) measurements at different scan rates were conducted. As shown in Figure S7, the LR‐F electrode exhibits higher fitting slopes than LR during both charge and discharge processes, indicating enhanced Li^+^ diffusion kinetic. Moreover, Electrochemical Impedance Spectroscopy combined with Distribution of Relaxation Times (DRT) analysis was performed to probe the interfacial and bulk transport processes. After 30 cycles, LR‐F shows substantially reduced charge transfer (*R_ct_*) and solid‐state diffusion resistances (*R_d_*) compared with LR (Figure S8). The decreased *R_ct_* is attributed to the increased exposure of electrochemically active (010) facets, which promotes interfacial Li^+^ migration and charge transfer. Meanwhile, the attenuated DRT peaks associated with interfacial and bulk Li^+^ diffusion processes demonstrate accelerated Li^+^ transport, resulting from the synergistic effects of fluorine‐induced local coordination regulation and optimized particle architecture. These results further validate the improved rate capability of the LR‐F electrode.

Since Li^+^ transport and electrode kinetics are not only governed by crystallographic diffusion pathways but also by the microstructural features of secondary particles, we examined further how F incorporation affects particle compaction, electronic conductivity, and internal porosity. A powder resistivity and compaction density measurement system (PRCD1100, Initial Energy Science & Technology; an instrument schematic is shown in Figure S9) was employed to systematically characterize the compaction density and electronic conductivity of the LR and LR‐F samples. The results are presented in Figure 3c and Table S2. Under low applied pressure (≈ 10 MPa), noticeable differences in compaction density and electronic conductivity were observed. Specifically, LR exhibits a lower compaction density (2.06 g cm^−3^) and electronic conductivity (4.7 µs cm^−1^) compared to LR‐F (2.36 g cm^−3^ and 9.9 µs cm^−1^, respectively). These differences reflect variations in secondary particle stacking, indicative of inter‐particle heterogeneity. Such heterogeneity arises from differences in particle morphology, contact points, and packing arrangement, which collectively govern particle interactions and macroscopic packing behavior. After applying a pressure of ≈270 MPa, LR and LR‐F exhibit comparable compaction densities (2.83 and 2.84 g cm^−3^, respectively), indicating similar macroscopic packing. Nevertheless, LR‐F maintains a higher electronic conductivity (65.1 vs 43.3 µs cm^−1^), pointing to intrinsic differences in intra‐particle architecture within the secondary particles.

To further confirm the difference in internal compactness between LR and LR‐F samples, synchrotron radiation small‐angle X‐ray scattering (SAXS) was employed to characterize the internal pore structure. SAXS analyzes the X‐ray scattering intensity across specific scattering vector ranges to compare porosity, with lower intensity corresponding to smaller pore volume and higher compactness [27]. The SAXS scattering intensities of LR and LR‐F were compared (Figure 3d), and the scattering curves were fitted using IGOR Pro software to establish a quantitative model correlating scattering intensity with pore volume (Figure 3d, inset). LR exhibits higher scattering intensity than LR‐F within the q = 0.01‐0.06 Å^−1^ range (corresponding to real‐space dimensions of 20–100 nm), and the relative porosity obtained through fitting is also higher than that of LR‐F (16.8% vs 0.4%), indicating a greater abundance of pore structures in LR secondary particles. This result is consistent with the conductivity differences and further corroborates that fluorine incorporation reduces porosity within secondary particles.

To evaluate whether the enhanced internal compactness of LR‐F secondary particles also translates into improved mechanical stability, the force performance of a single particle was measured by a high‐precision nano‐control force and displacement sensors based on a single particle force tester (Figure S10). By analyzing the characteristic parameters of the pressure‐displacement curve (Figure 3e), a comprehensive comparison was made between LR and LR‐F samples in terms of particle compressive resistance and structural stability. The pressure‐displacement curve exhibits three distinct stages. During the initial compression stage, particles undergo elastic‐plastic deformation, leading to a gradual increase in the curve. As pressure rises to the structural load‐bearing limit, the curve drops suddenly at the crushing point, with the corresponding force defined as the crushing force. The indenter then continues to descend, causing the particle structure to disintegrate and the curve to decline sharply. Once the indenter contacts the substrate, the curve resumes increasing. Based on this testing mechanism, differences in mechanical properties between the two samples can be directly quantified by comparing their respective crushing forces [28, 29]. The test results are presented in Figure 3f and Table S3 and S4. The average crushing force of the LR‐F samples (12.02 mN) is significantly higher than that of the LR samples (3.06 mN), indicating that F incorporation enhances the internal compactness of secondary particles, thereby improving their compressive strength and overall mechanical properties. This observation is consistent with previous findings and confirms that fluorine facilitates a synergistic enhancement of both particle architecture stability and mechanical stability by regulating particle morphology.

To elucidate the practical implications of secondary particle internal compactness at the electrode level, we prepared and evaluated both LR and LR‐F electrodes under identical processing conditions. As demonstrated in Table S5, while both electrodes share a similar coating thickness, the LR‐F electrode delivers significantly higher areal mass loading and compaction density. Electrode compaction density denotes the mass per unit electrode volume, indicating improved volumetric packing efficiency in LR‐F. Although this difference appears limited at the coin‐cell scale, it is expected to be significantly amplified during industrial scale‐up, thereby critically influencing the volumetric energy density of full cells.

### 2.3 Modulating Local Covalent Environments to Enhance Anionic Redox Reversibility

To determine whether the introduced F^−^ ions occupy lattice sites originally held by other elements, neutron powder diffraction (NPD) was conducted to evaluate the site occupancy, as shown in Figure 4a and Table S6. Rietveld refinement reveals that F^−^ ions preferentially occupy oxygen sites, with a refined F fractional occupancy of 0.0036 for these sites. After normalization, this occupancy corresponds to an F atomic ratio of approximately 0.72 mol%. This NPD‐derived F content is further supported by sequential ion chromatography (IC) analysis, which differentiates the surface‐residual and lattice‐associated fluorine fractions. Specifically, IC analysis reveals a surface residual F content of 0.13 mol% and a lattice‐incorporated F content of 0.60 mol% (Table S7). The comparable F contents obtained from NPD refinement and IC quantification provide independent evidence for substitutional F incorporation into the host lattice, thereby supporting the F‐induced modulation of the local covalent environment. To elucidate how such local structural evolution influences electrochemical properties, the initial electrochemical performance of LR and LR‐F cathodes was systematically evaluated within 2.0‐4.8 V at 0.05C. As shown in Figure 4b (left), LR‐F delivers an initial Coulombic efficiency of 84.9%, representing a 6.8% improvement over LR, while maintaining a comparable charge capacity (309.8 vs 312.3 mA h g^−1^). The corresponding differential capacity (dQ/dV) curves (Figure 4b, right) show an attenuated oxidation peak at ∼ 4.5 V for LR‐F, accompanied by a shortened high‐voltage oxygen redox plateau, indicating suppressed lattice oxygen activation. Considering that excessive oxygen oxidation is typically associated with irreversible structural evolution (e.g., O─O dimer formation and oxygen release) [30], this observation further implies that the corresponding irreversible lattice oxygen loss is mitigated. Direct evidence is provided by in situ online electrochemical mass spectrometry (OEMS, Figure 4b, bottom) and associated charge‐discharge curves (Figure S12), which reveal substantially reduced O_2_ evolution in LR‐F during the first charge (0.2C, cutoff voltage of 4.8 V), confirming suppressed irreversible lattice oxygen loss. To further probe oxygen redox activity, O *K*‐edge soft X‐ray absorption spectroscopy (sXAS) spectra collected in fluorescence yield (FY) mode were analyzed (Figure 4c). The integrated intensity in the 525–534 eV range reflects the relative population of unoccupied O 2*p* states above the Fermi level (i.e., oxygen holes) [31, 32]. At a deeply charged state (4.8 V), LR exhibits a slightly higher integrated intensity than LR‐F (Figure 4c, inset), indicating differences in oxygen redox behavior between the two materials. This observation suggests that fluorine incorporation may influence lattice oxygen oxidation. Notably, the two materials exhibit comparable charge capacities, implying that differences in oxygen redox behavior may be compensated by variations in cationic redox contributions. To clarify this charge compensation mechanism, Ni/Mn *K*‐edge X‐ray absorption near‐edge structure (XANES) spectra were further analyzed (Figure 4d,e). Due to the strong electronegativity of fluorine, the initial transition‐metal valence states in LR‐F are lower. Upon charging to high voltage, however, the Ni/Mn valence states of both materials converge to similar levels, and their reduction behaviors during discharge are also comparable (Figure 4d,e, inset). These results indicate that LR‐F undergoes a larger variation in Ni/Mn oxidation states during cycling, implying enhanced participation of cationic redox. Considering the nearly identical charge capacity, this suggests a redistribution of the charge compensation mechanism, which may be associated with differences in anionic redox behavior between the two materials.

**FIGURE 4:**
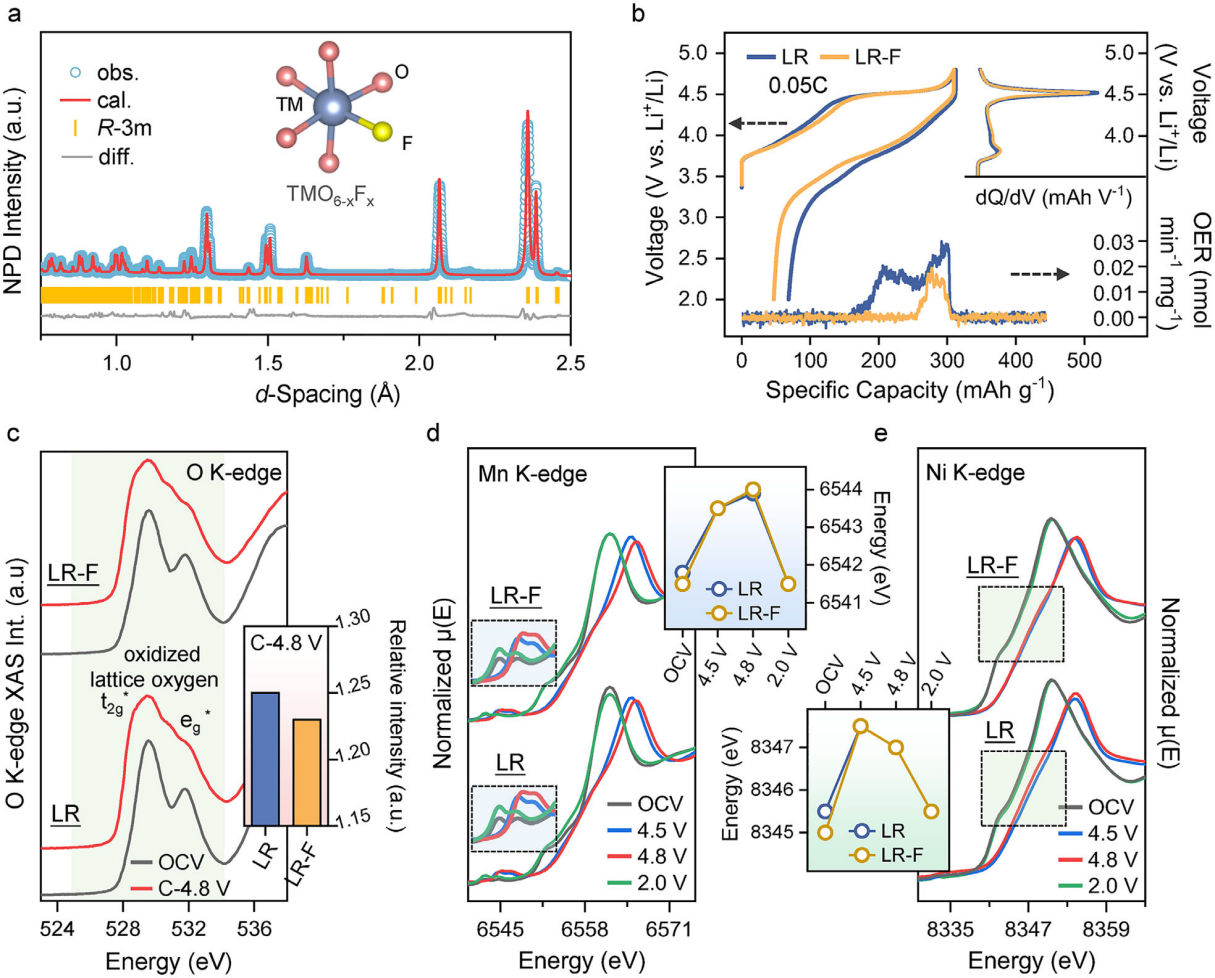
Effect of fluorine incorporation on crystal structure, electrochemical behavior and electronic structure evolution. (a) Neutron powder diffraction (NPD) pattern and Rietveld refinement results of the LR‐F cathode. (b) Initial charge–discharge curves and corresponding differential capacity (dQ/dV) curves measured at 25 °C, 0.05C, and 2.0–4.8 V. Insert: The online electrochemical mass spectrometry (OEMS) results during the first charge at 0.2C, OCV‐4.8 V. (c) O *K*‐edge sXAS spectra collected in FY mode, details of the normalization procedure are provided in the (Figure S11). The concentration of oxygen holes can be derived from the shaded region (525–534 eV) in the spectra. For each compound (LR and LR‐F), the red C‐4.8 V spectrum and the corresponding black OCV spectrum were integrated over this shaded energy region, and then the ratio of the “red” and the “black” integrals was taken. This is the “relative intensity” plotted in the inset of Figure 4c. Evolution of normalized XANES spectra of (d) Mn and (e) Ni *K*‐edge during the initial charge and discharge process. Insert: Half‐height changes during in operando XANES at the Mn and Ni *K*‐edge.

Given the strong coupling between oxygen redox behavior and Li^+^ re‐intercalation, the Li^+^ reinsertion behavior was further investigated using ^7^Li solid‐state nuclear magnetic resonance (NMR) spectroscopy with a 2D projection based on the magic‐angle turning phase‐adjusted spinning sideband (pj‐MAT PASS) technique. As shown in Figure 5a, two resonances are observed at ∼710 ppm and ∼1500 ppm, corresponding to Li in the alkali metal layer (AM_Li_) and transition metal layer (TM_Li_), respectively [33]. Existing research has shown that the deconvoluted spectral intensity can serve as a semi‐quantitative indicator of the relative concentration of Li^+^ [34]. Based on this, the deconvoluted area of TM_Li_ peaks for the LR and LR‐F samples discharged to 2.0 V was compared. The integration results are depicted in a pie chart, with the percentages representing the TM_Li_ content relative to their initial levels (open‐circuit voltage, OCV state). Only 63.1% of Li^+^ ions are reversibly reinserted in LR, whereas the value increases to 86.5% in LR‐F. These differences highlight that Li^+^ re‐intercalation is strongly linked to electrochemical reversibility in LRLO cathodes. Rather than an isolated kinetic process, it is coupled with the availability of lithium sites, which is governed by lattice oxygen redox evolution and associated TM migration. Accordingly, Li^+^ re‐intercalation can be viewed as a sensitive probe of the coupled oxygen redox and TM migration processes. From this perspective, fluorination is expected to modulate oxygen redox activity and its structural consequences, thereby affecting Li^+^ transport and reinsertion behavior. Further spectroscopic and theoretical analyses are provided in the following sections to clarify the nature of oxygen redox, the formation of O–O species, and their role in structural evolution and Li^+^ transport kinetics.

**FIGURE 5:**
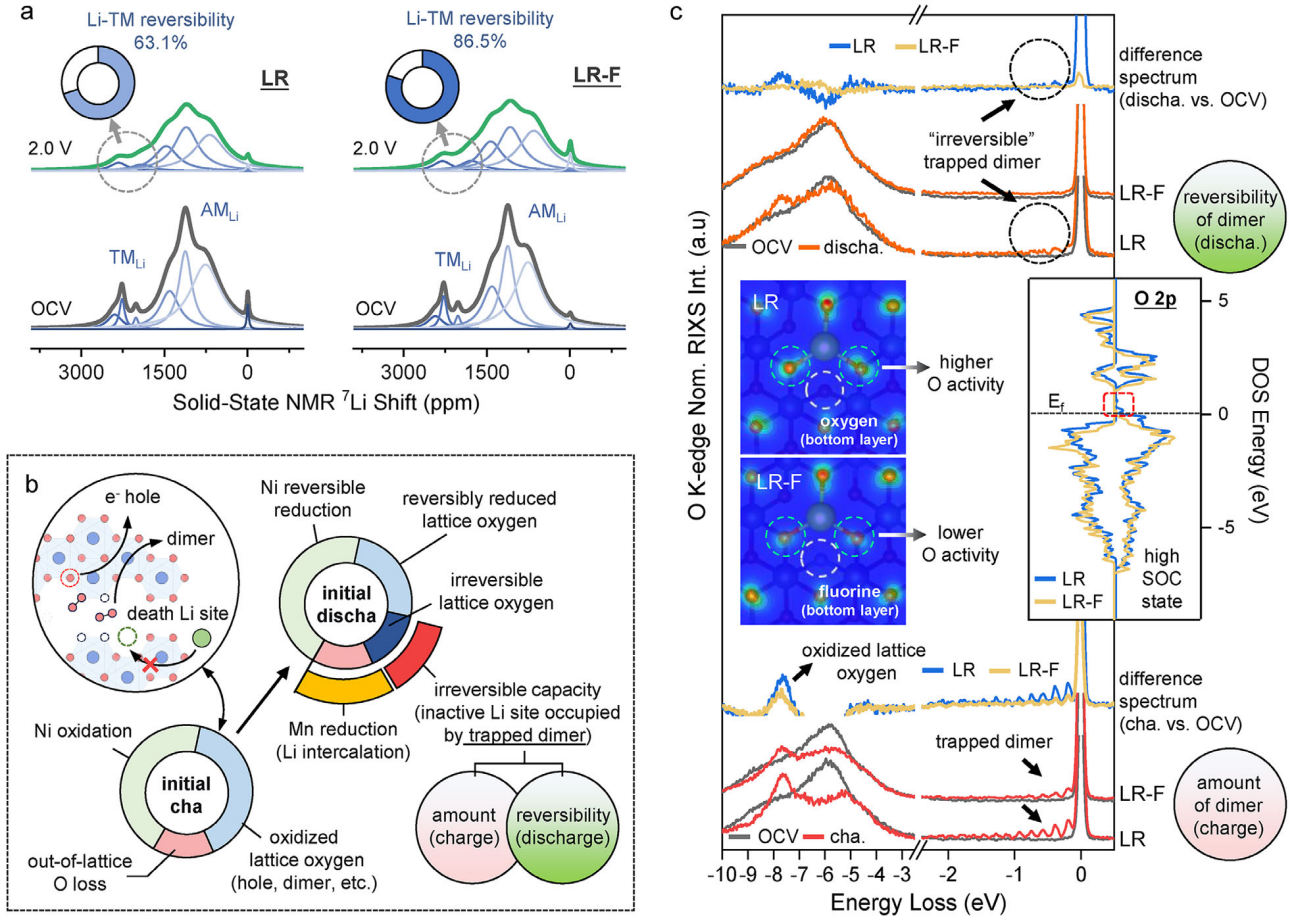
Reversibility of Li deintercalation and the behavior of lattice oxygen. (a) Solid‐state NMR (^7^Li) spectra of LR and LR‐F. (b) Schematic illustrating of lattice oxygen behavior and the origin of irreversible capacity during the initial charge and discharge. (c) O *K*‐edge high‐resolution RIXS spectra of LR and LR‐F, recorded at an excitation energy of 531 eV in the OCV state and after the first discharge to 2.0 V. The corresponding difference spectra (relative to OCV) highlight trapped oxygen dimers (∼0 eV energy loss) and oxidized lattice oxygen (∼7 eV). Inset: The oxygen‐projected density of states was calculated for two samples at high SOC state.

To elucidate the origin of the observed capacity differences, a schematic illustration is provided (Figure 5b). During the initial charge, the capacity contributions can be primarily attributed to two sources: the oxidation of the TM (Ni) and the oxidation of lattice oxygen [35]. The latter can be further subdivided into two components: oxygen lost due to escape from the lattice and oxidized oxygen species that remain confined within the lattice, including oxygen electron holes and oxygen dimers [7]. In the initial discharged state, the redox reactions of Ni are generally considered to be highly reversible with negligible loss. Therefore, the observed discrepancy in capacity is primarily governed by the redox behavior of lattice oxygen. The charge deficit resulting from oxygen release can be compensated by the reduction of Mn [17], effectively offsetting the associated capacity loss, but not all oxidized oxygen species behave in the same manner. Those retained within the lattice can be further classified based on their discharge behavior into reversible (reducible) and irreversible (e.g., trapped oxygen dimers) types. The irreversible species give rise to persistent capacity loss that cannot be compensated by Mn reduction, as they remain trapped within the crystal lattice, occupying sites that would otherwise be available for Li^+^ re‐intercalation and thereby hindering it. Consequently, the presence of trapped oxygen species hinders Li^+^ reinsertion, which in turn limits the associated charge‐compensating reduction of Mn. Therefore, the overall irreversibility is governed by both the extent of oxygen activation during charge and the reversibility of oxygen redox during discharge.

To directly probe lattice oxygen behavior, high‐resolution O *K*‐edge resonant inelastic X‐ray scattering (RIXS) spectra were acquired at OCV, upon charging to 4.8 V (cha.), and upon discharging to 2.0 V (discha.), along with corresponding difference spectra (Figure 5c). The ∼7 eV energy loss feature is assigned to oxidized lattice oxygen, while the vibrational progression near 0 eV is associated with O_2_ molecule‐like trapped O─O dimer (trapped dimer). Compared with LR, LR‐F exhibits significantly weaker signals for both oxidized oxygen and trapped dimers, indicating suppressed oxygen activation and improved oxygen redox reversibility. DFT calculations were performed to further verify the aforementioned conclusion. The electronic structures of the pristine LR, LR‐F, and highly delithiated counterparts (Li content = 0.08) were systematically investigated, the corresponding crystal structure is shown in Figure S13. The projected density of states (PDOS) derived from O 2*p* orbitals of delithiated LR and LR‐F systems are presented in the middle panels of Figure 5c (middle panels). For the deeply delithiated LR system, distinct O 2*p* orbital contributions appear in the energy region of 0–1.2 eV above the Fermi level, whereas this phenomenon is absent in its pristine state (Figure S14). This verifies the occurrence of oxygen oxidation under deep delithiation (red dashed box). By contrast, the oxygen neighboring F in the LR‐F system exhibit negligible O 2*p* density of states within the identical energy region. Such a distinct difference reveals that the delithiated LR material undergoes more significant oxygen 2*p* hole formation and hence delivers enhanced oxygen redox. According to the mechanism proposed by Bruce et al. [36], oxygen redox in materials with specific cationic ordering (e.g., honeycomb or ribbon superstructures) involves the formation of electron holes localized on oxygen atoms within distinct coordination environments. In the LR material, these holes are primarily associated with oxygen atoms coordinated by two Mn ions (O‐Mn_2_ configurations). The origin of oxygen redox behavior revealed by PDOS analysis can be visualized via projected charge density distribution within the energy region of 0–1.2 eV above the Fermi level. As shown in the inset of Figure 5c left and Figure S15, the projected charge density mapped from Ef to E_f_ + 1.2 eV confirms that F incorporation reduces the electronic contribution of adjacent oxygen, thereby suppressing the oxygen 2*p* electron hole formation. This effect suppresses the excessive oxidation of lattice oxygen during charging. The excessive oxidation of LR material during delithiation would lead to lattice oxygen loss. The consistence between DFT calculations and experimental observations supports the conclusion that fluorination effectively modulates oxygen redox activity by regulating local covalent bonding environments.

Beyond suppressing excessive oxygen activation, fluorination also enhances the reversibility of oxygen redox by regulating the coupling between TM and lattice oxygen. When oxygen is over‐oxidized, the stability of the Mn‐O framework is compromised, leading to partial bond breaking and weakened TM‐O hybridization. This structural destabilization lowers the migration barrier of TM, facilitating their migration from lattice sites and creating undercoordinated oxygen species [37]. This process reflects a strong coupling between electronic instability (oxygen over‐oxidation) and structural degradation (TM migration and bond rupture). These highly reactive oxygen species tend to recombine and form trapped O─O dimers, which represent a major irreversible pathway during cycling [18]. The accumulation of such dimers not only consumes active oxygen redox centers but also occupies Li sites and blocks Li^+^ diffusion channels, thereby impeding Li^+^ reinsertion and resulting in significant irreversible capacity loss. In contrast, fluorination introduces strong Mn‐F interactions that reinforce the local bonding environment and stabilize the TM‐anion framework against over‐oxidation‐induced degradation. By suppressing Mn‐O bond breaking and subsequent transition metal migration, fluorination effectively reduces the formation of O─O dimers. Meanwhile, the preserved electronic coupling between TM and oxygen facilitates more efficient electron back‐donation to oxidized oxygen species during discharge, promoting the reversible reduction of O─O species. Furthermore, in the ∼3.3 V region (Figure S16), galvanostatic intermittent titration technique (GITT) analysis reveals that LR exhibits a markedly lower Li^+^ diffusion coefficient than LR‐F, indicating sluggish Li^+^ transport kinetics at low voltages. This reduced diffusivity implies the presence of residual unreduced O─O dimers in LR, which suppress Li^+^ transport and further corroborate the above conclusion.

### 2.4 Cross‐Scale Structural Robustness and Long‐Term Cycling Stability

To investigate the effect of fluorine incorporation on the long‐term cycling stability of Li‐rich cathodes, a comparative analysis of LR and LR‐F was conducted, focusing on electrochemical behavior and local structure evolution. Figure 6a presents the charge‐discharge profiles and corresponding differential capacity (dQ/dV) curves after 300 cycles. Both electrodes exhibit characteristic voltage decay, as evidenced by the continuous decline in discharge voltage and the evolution of dQ/dV profiles, which include peak broadening, intensity attenuation, and potential shifts. These features are typically associated with increased polarization, irreversible ARR, and TM migration [38]. Obviously, these degradation features are markedly suppressed in LR‐F. Notably, the LR‐F electrode maintains a stable low‐voltage redox feature (∼3.0 V) without observable peak shift during prolonged cycling, indicating inhibited spinel‐like phase evolution and a preserved local Mn coordination environment [39]. The strengthened TM–O covalency further stabilizes Mn^3+^/Mn^4+^ redox activity, contributing to a more reversible charge‐compensation process coupled with lattice oxygen redox. This stabilized electrochemical signature suggests enhanced electronic robustness of Mn–O interactions, which effectively mitigates structural degradation and preserves the local coordination environment during cycling. The cycling performance shown in Figure 6b (25 °C) and Figure 6c (55 °C) further highlights the beneficial role of F incorporation. At 25 °C, the LR cathode delivers an initial capacity of 208.2 mA h g^−1^ at 0.5C but retains only 65.3% after 300 cycles. In contrast, LR‐F exhibits a comparable initial capacity (208.3 mA h g^−1^) while maintaining a high‐capacity retention of 88.8% after 950 cycles. Meanwhile, as shown in Figure S17 and Table S8, the LR‐F electrode exhibits a significantly lower average discharge voltage decay rate (0.70 mV cycle^−1^) over the 1–300 cycle range compared with the pristine LR electrode (1.66 mV cycle^−1^). Furthermore, increasing fluorine content correlates positively with electrochemical performance (Figure S18, showing coulombic efficiency and cycling stability). However, excessive fluorine incorporation leads to performance degradation, suggesting that an optimal fluorine concentration exists to balance structural regulation and electrochemical stability. Table S9 summarizes the relationship between fluorine content, morphological evolution, internal compactness, and electrochemical performance. These results further indicate that fluorine modifies the surface energy of primary particles, thereby regulating their morphology, enhancing particle internal compactness, and ultimately leading to improved electrochemical performance. GITT analysis of the LR‐F cathode reveals enhanced Li^+^ diffusion at low voltages after 100 cycles (Figure 6b, inset), suggesting that fluorine incorporation facilitates the reduction of oxidized lattice oxygen and thereby promotes reversible Li^+^ reinsertion during discharge. At elevated temperature (55 °C), the LR cathode delivers an initial discharge capacity of 262.8 mA h g^−1^, with 88.2% retention after 180 cycles. By comparison, the LR‐F cathode achieves a higher initial discharge capacity of 268.8 mA h g^−1^ and retains 94.0% under identical conditions, demonstrating superior cycling stability.

**FIGURE 6:**
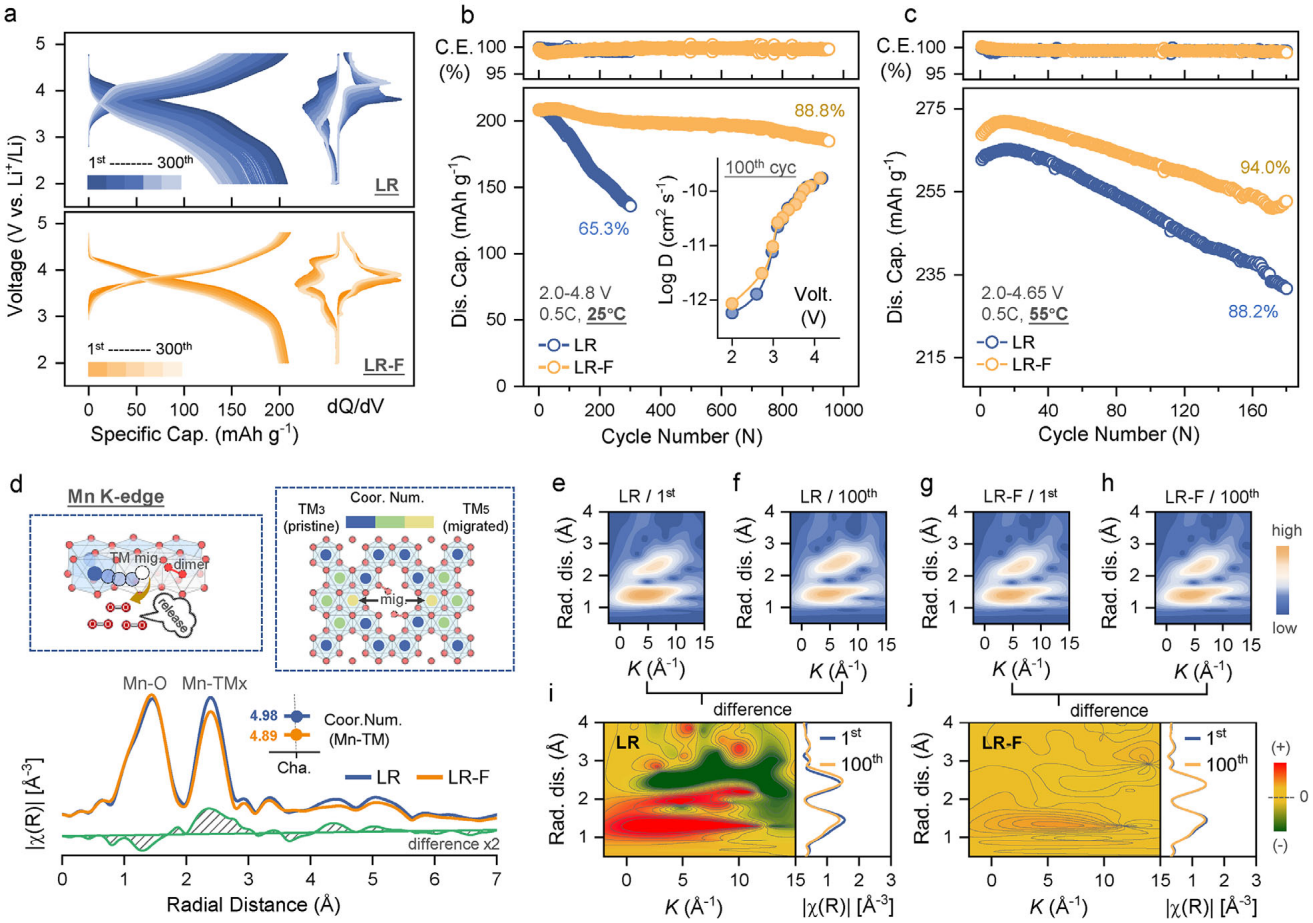
Differences in long‐term cycling stability and crystal structure degradation between LR and LR‐F cathodes. (a) Charge‐discharge curves and corresponding dQ/dV curves from different cycles at 25 °C, 0.5C, 2.0–4.8 V. (b) Cycle performance at 25 °C, 0.5C, 2.0–4.8 V. (c) Cycle performance at 55 °C, 0.5C, 2.0–4.65 V. (d) Mn *K*‐edge EXAFS data obtained at the fully charged state (4.8 V). Insert: Mn‐TM coordination and corresponding structural model. Wavelet transform EXAFS spectra of LR at (e) first and (f) 100 cycles, and of LR‐F at (g) first and (h) 100 cycles, obtained at the fully charged state (4.8 V). The EXAFS and corresponding difference spectra from WT‐EXAFS spectra of (i) LR and (j) LR‐F cathodes.

To correlate the improved electrochemical performance with structural stability, extended X‐ray absorption fine structure (EXAFS) analysis was conducted to compare local structural evolution in the two cathodes. The Mn *K*‐edge EXAFS spectra (Figure 6d, phase shift uncorrected) exhibit two main features: the peak at ∼1.6 Å corresponds to the first‐shell Mn‐O coordination, while the peak at ∼2.4 Å arises from second‐shell Mn‐TM coordination. As shown in the Figure 6d (inset), oxygen loss leads to attenuation of the Mn‐O peak, whereas in‐plane TM migration results in an enhanced Mn‐TM peak intensity [40, 41]. After 100 cycles at full charge, the LR cathode displays a markedly weakened Mn‐O peak and a significantly intensified Mn‐TM peak, indicating severe irreversible ARR, substantial oxygen loss, and consequent TM migration. In contrast, LR‐F retains a stronger Mn‐O peak and a weaker Mn‐TM peak, reflecting improved reversibility of ARR and suppressed TM migration. To further quantify changes in the TM coordination environment, the EXAFS data are fitted (Figure 6d, inset, detailed parameters in Figure S19). The results reveal that the TM ions in the LR‐F sample possess a lower coordination number (N). These results indicate that F incorporation effectively suppresses distortion of the Mn local coordination environment, thereby preserving the integrity of the layered lattice.

Furthermore, wavelet transform (WT) analysis was performed on Mn *K*‐edge EXAFS spectra collected at full charge following the first and 100 cycles. As shown in Figure 6e,f, the WT spectrum of the LR cathode exhibits a pronounced attenuation of the oxygen coordination signal accompanied by a clear enhancement of the TM coordination signal after 100 cycles, indicative of accelerated oxygen loss and intensified TM migration. In contrast, the LR‐F cathode shows only a slight decrease in the oxygen coordination signal and negligible change in the TM coordination signal (Figure 6 g,h). The corresponding WT‐EXAFS and difference spectra further highlight the evolution of the Mn‐O and Mn‐TM coordination environments in LR and LR‐F (Figures 6i,j). Collectively, these results demonstrate that LR‐F undergoes significantly less structural distortion than LR after prolonged cycling, confirming that fluorine incorporation enhances the structural stability of the framework.

To further correlate the structural evolution with electrochemical degradation, multiscale structural changes (from atomic to particle level) were evaluated by disassembling coin cells after 200 cycles. The recovered electrodes were analyzed using sXRD to probe phase transformation and time‐of‐flight secondary ion mass spectrometry (TOF‐SIMS), in combination with FIB‐SEM, to map the spatial distribution of Li^+^. Rietveld refinement of the sXRD patterns (Figure 7a, Table S10 and S11) reveals a higher fraction of spinel phase in LR (4.8%) than in LR‐F, where no detectable spinel phase is observed in LR‐F, indicating more severe structural transformation in LR compared with LR‐F. Meanwhile, TOF‐SIMS maps (Figure 7b) show that LR particles exhibit pronounced internal Li heterogeneity in the charged state, with Li‐rich domains (associated with inert/distorted rock‐salt phases, referred to as the DRX phase) accumulating near the surface and gradually propagating inward. This behavior indicates significant loss of active lithium and hindered Li^+^ transport. In contrast, the more compact LR‐F particles display a homogeneous Li distribution with only minor surface Li enrichment, suggesting improved Li^+^ (de)intercalation reversibility.

**FIGURE 7:**
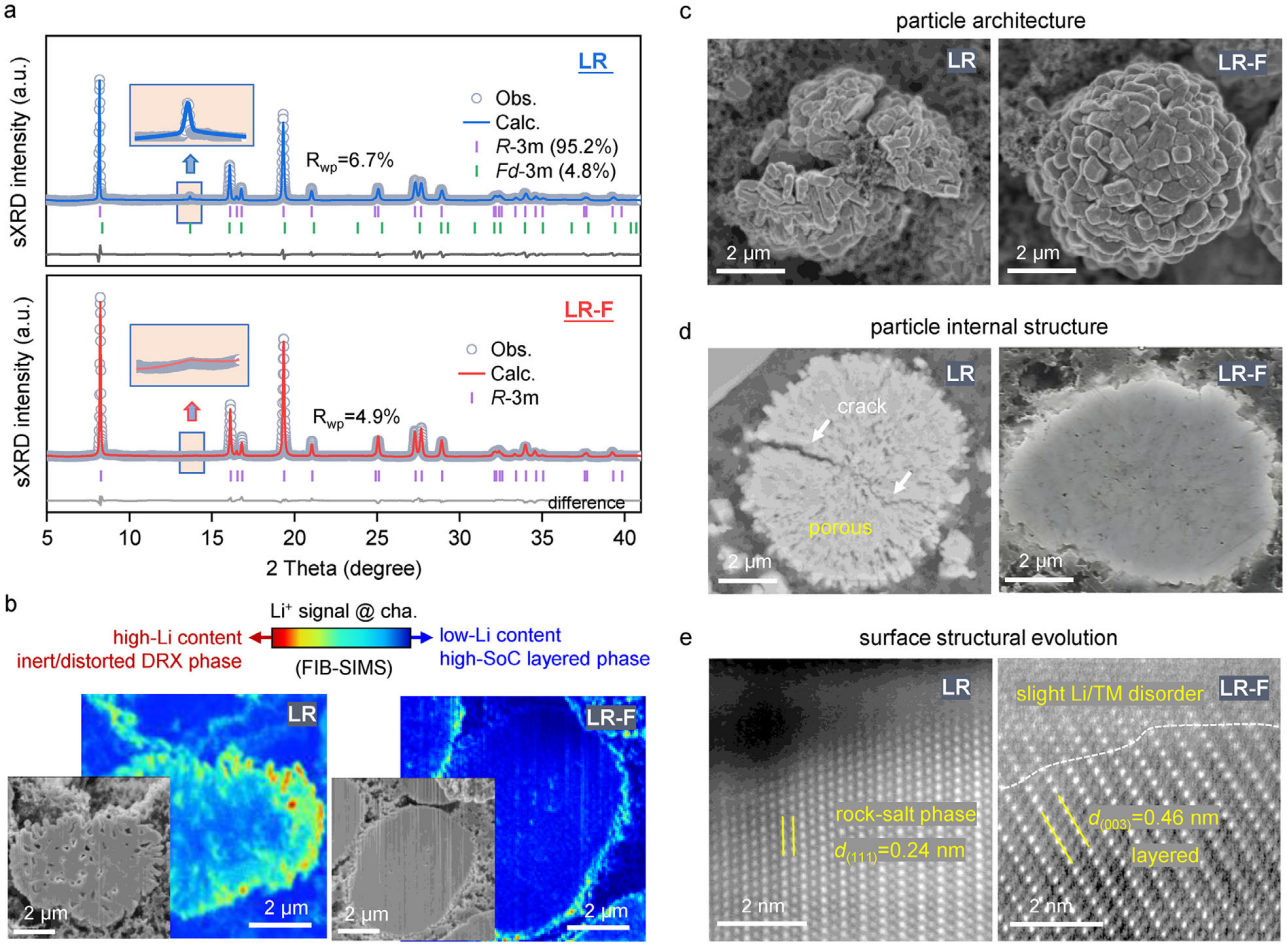
Structural and morphological evolution of LR and LR‐F cathodes after long‐term cycling. (a) sXRD patterns and refined results of electrode sheets after 200 cycles at 25 °C, 0.5C, 2.0–4.8 V. (b) TOF‐SIMS maps of the normalized elemental distribution of ^7^Li in charged cathodes, obtained from half‐cells after 200 cycles at 25 °C, 0.5C, 2.0–4.8 V. SEM image of (c) the secondary particle and (d) the cross‐sectional view after 300 cycles, 25 °C, 0.5C, 2.0–4.8 V. (e) HAADF‐STEM images of both cathodes viewed along the [010] zone axis after 300 cycles, 25 °C, 0.5C, 2.0–4.8 V.

Particle integrity was further evaluated after 300 cycles. SEM images (Figure 7c) reveal severe fragmentation in LR particles, whereas LR‐F particles largely retain their structural integrity. Cross‐sectional analysis (Figure 7d) shows extensive intergranular cracks penetrating LR secondary particles, while only nanoscale pores are observed in LR‐F, confirming its superior mechanical robustness. At the atomic scale, high‐angle annular dark‐field scanning transmission electron microscopy (HAADF‐STEM) imaging (Figure 7e) reveals distinct surface reconstruction behaviours between the two samples. For the LR cathode, irreversible oxygen loss and extensive transition‐metal migration induce severe surface reconstruction, as evidenced by the formation of a pronounced surface rock‐salt phase with a *d*_(111)_ spacing of 0.24 nm. In contrast, the LR‐F sample maintains superior structural integrity, preserving a well‐defined layered structure with a characteristic *d*_(003)_ spacing of 0.46 nm and only minor Li/TM disorder. These observations further demonstrate the stabilizing effect of F incorporation on the layered lattice structure. In summary, F incorporation enhances the long‐term cycling stability of LRLO cathodes through multiscale effects: (i) structurally, it suppresses the layered‐to‐rock‐salt phase transformation, preserving Li^+^ diffusion pathways; (ii) morphologically, compact internal structure mitigates electrolyte penetration and crack formation, thereby reducing active material loss; and (iii) interracially, it stabilizes the surface oxygen framework and improves the reversibility of ARR.

Furthermore, comprehensive compositional and interfacial analyses consistently indicate that the enhanced electrochemical performance of LR‐F is fundamentally governed by intrinsic structural and morphological regulation rather than surface passivation‐related effects. Specifically, TOF‐SIMS combined with FIB‐SEM (Figure S20) and IC analyses indicate the presence of fluorine‐containing residues on the material surface, likely in the form of LiF. However, this LiF residue is neither continuous nor uniformly distributed across the surface, and therefore is not expected to function as an effective protective coating layer. Importantly, despite the pronounced divergence in electrochemical performance between the two samples (Figure S21a), TOF‐SIMS analysis of the cycled electrodes reveals a highly comparable extent of interfacial electrolyte decomposition, as evidenced by the similar absolute signal intensities of characteristic secondary ion fragments associated with electrolyte decomposition products (Figures S21b and S21c). These results collectively suggest that neither a protective surface coating nor a reduction in interfacial side reactions is the dominant origin of the performance enhancement. Instead, the superior cycling stability of LR‐F is primarily attributed to the cross‐scale synergistic effects of F incorporation, which optimizes particle compactness and local covalent bonding within the bulk structure.

### 2.5 Practical Viability and Enhanced Thermal Safety in Ah‐Level Full Cells

To further validate the practical applicability of the LR‐F cathode, pouch‐type full cells were assembled to evaluate long‐term cycling stability. The key parameters for the electrode fabrication are summarized in Table S12. In terms of electrochemical performance, the graphite (Gr)||LR‐F single‐layer pouch‐type full cell (0.5 Ah) retained 96.3% of its capacity after 1500 cycles at charge/discharge rates of 0.5/1.0C within the industrially relevant voltage window of 2.5–4.75 V (Figure 8a). Meanwhile, the Gr||LR‐F multilayer pouch‐type full cell (2.0 Ah) exhibited a capacity retention of 94.8% after 3000 cycles at 1.0/2.0C within a voltage range of 2.5–4.65 V (Figure 8b). To further assess the practical electrochemical stability of LR‐F cathodes under Ah‐level conditions, the charge–discharge profiles and median discharge voltage evolution of Gr||LR‐F pouch cells were systematically evaluated (Figure S22). Both the 0.5 Ah single‐layer and 2.0 Ah multi‐layer pouch cells maintained highly stable median discharge voltages with negligible decay after 1500 and 3000 cycles, respectively (Figures S22a and S22b). Moreover, the charge–discharge profiles at selected cycles exhibited nearly complete overlap (Figures S22c and S22d), indicating suppressed polarization and highly reversible electrochemical behavior. These results demonstrate that the LR‐F cathode not only delivers enhanced performance in half‐cell systems but also exhibits outstanding long‐term durability in Ah‐level multilayer pouch‐type full cells. Regarding thermal runaway risk assessment, accelerating rate calorimetry (ARC) was performed, a widely used technique to probe self‐heating reactions under adiabatic conditions. Two key characteristic temperatures are defined: *T_onset_*, the onset temperature of self‐heating, and *T_TR_*, the temperature at which thermal runaway is triggered [42, 43, 44]. The ARC results (Figure 8c,d) show that the Gr||LR‐F pouch‐type full cell (1.5 Ah) exhibits a markedly higher *T_onset_* (137 °C) than the Gr||LR full cell (114 °C). The *T_TR_* of the LR‐F cell also reaches 165 °C, exceeding that of the LR cell (150 °C), and is accompanied by a significantly lower temperature rise rate (*dT/dt*) during thermal runaway. These results demonstrate that fluorine incorporation effectively elevates the thermal stability threshold of the full cell and mitigates thermal runaway risk.

**FIGURE 8:**
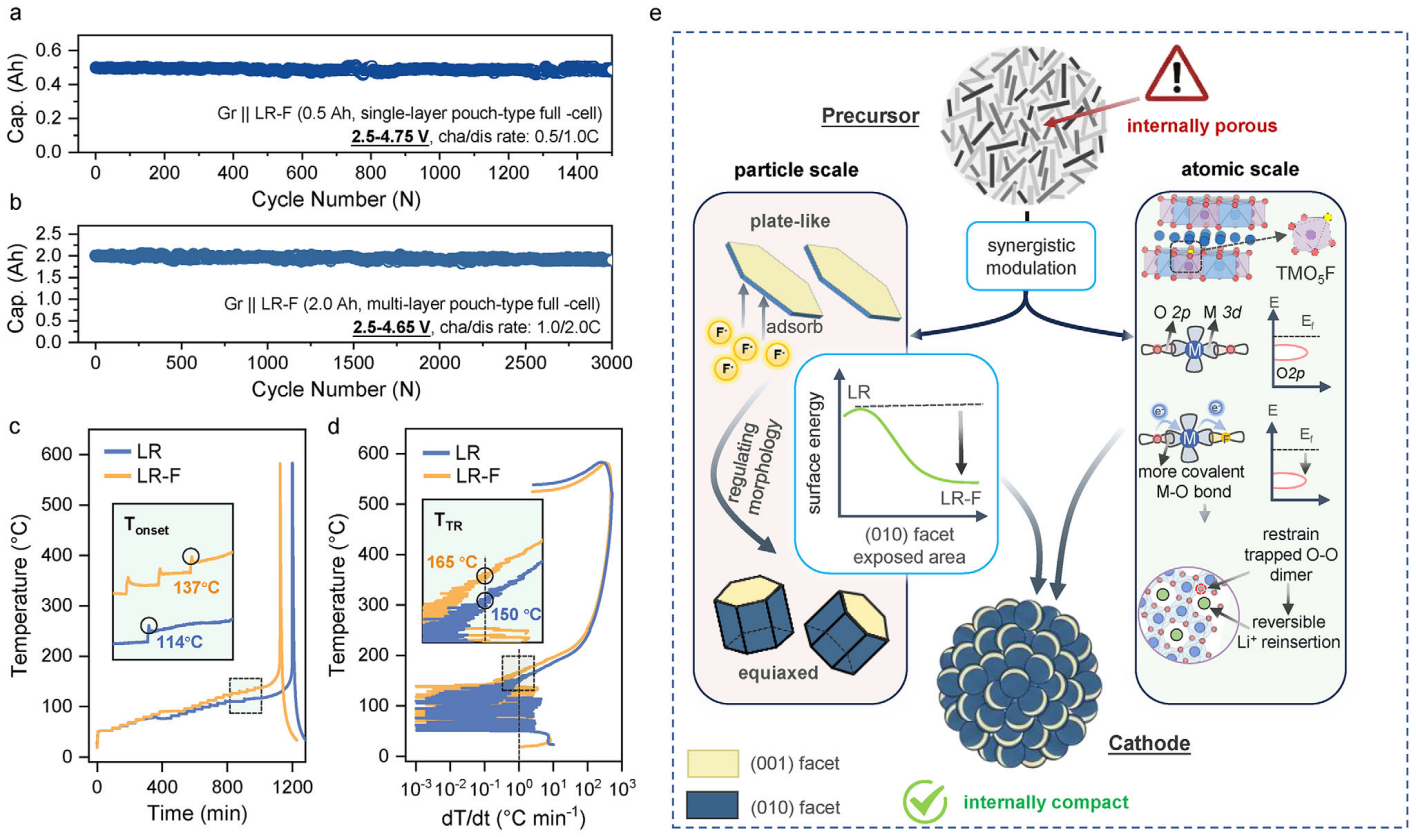
Cycling performance and thermal runaway risk assessment of full‐cell. Capacity at different cycle numbers for (a) the Gr||LR‐F single‐layer pouch‐type full‐cell (0.5 Ah, 2.5–4.75 V) and (b) the Gr||LR‐F multi‐layer pouch‐type full‐cell (2.0 Ah, 2.5–4.65 V). (c) Temperature versus time plot and (d) temperature versus heating rate plot of charged Gr||LR and Gr||LR‐F pouch cells during thermal runaway testing with ARC. (e) Schematic illustration of the regulatory effects of F at the particle and atomic scale.

Overall, the LR‐F cathode exhibits a favourable combination of high capacity, prolonged cycle life, and enhanced safety. Figure 8e schematically illustrates the fluorine‐induced synergistic regulation mechanism in LRLO cathodes. At the particle scale, fluorine modulates the surface energy of the (010) facet, increasing its exposure and transforming the primary particle morphology from plate‐like to equiaxed. This promotes dense packing of primary particles, enhances the compactness of secondary particles, and suppresses the inheritance of porous structures. At the atomic scale, fluorine is incorporated into the lattice to form an TMO_5_F configuration, which strengthens TM‐O covalency, suppresses excessive oxygen activation, and improves the reversibility of ARRs. This cross‐scale synergistic regulation not only resolves the structural instability associated with particle architecture in LRLO cathodes but also optimizes oxygen redox kinetics, ultimately leading to a comprehensive enhancement in electrochemical performance.

## 3 Conclusion

In this work, we establish a direct correlation between internal compactness and particle architectural stability, identifying it as a key descriptor of cathode structural integrity. Simultaneously, the capacity contribution and compensation mechanism of ARR are resolved, revealing that the extent of irreversible capacity loss is governed by both the degree of oxygen activation during charging and the reversibility of oxygen redox during discharging. Guided by these insights, a cross‐scale synergistic strategy (fluorine incorporation) is developed to reinforce particle architectural stability and enhance ARR reversibility.

At the particle scale, fluorine regulates the surface energies of crystallographic planes, suppressing the preferential exposure of (001) facets while promoting (010) facets. This thermodynamic transformation shifts primary particles from plate‐like to equiaxed morphologies, effectively eliminating precursor‐inherited porosity and enabling the formation of highly compact secondary particles. This structural modulation markedly enhances electrochemical performance, delivering an outstanding capacity retention of 88.8% after 950 cycles. Moreover, the increased exposure of (010) facets provide more accessible Li^+^ diffusion channels, reducing transport resistance and facilitating rapid Li^+^ migration along low‐energy‐barrier pathways. Consequently, the LR‐F cathode exhibits superior rate capability, achieving a high discharge capacity of 186.1 mA h g^−1^ at 5C, representing a significant improvement over the pristine counterpart. At the atomic scale, we reveal that irreversible capacity decay is primarily governed by irreversibly oxidized oxygen species (e.g., trapped oxygen dimers). Through fluorine incorporation, the TM‐O covalency is strengthened, which suppresses excessive oxygen activation, reduces trapped dimer formation, and stabilizes the lattice framework, thereby delivering an enhanced initial Coulombic efficiency of 84.9%.

Notably, this cross‐scale regulation strategy is further validated in practical graphite‐anode full‐cell configurations. At the Ah level, the assembled 0.5 Ah pouch cell exhibits excellent cycling stability at a high cut‐off voltage of 4.75 V, delivering a capacity retention of 96.3% after 1500 cycles. Under milder operating condition (4.65 V cut‐off), the 2.0 Ah full cell demonstrates unprecedented long‐term durability, sustaining 94.8% capacity retention over 3000 cycles. Overall, this cross‐scale synergistic strategy establishes a generalizable design principle for integrating multiscale structural regulation with anionic redox chemistry, unlocking the rational design of high‐performance, inherently safe LRLO cathodes for next‐generation energy storage.

## Author Contributions

**Yizhen Huang**: investigation, writing – original draft, data curation. **Yu Qiao**: conceptualization, methodology, software, data curation, supervision, resources, project administration, visualization, writing – review and editing. **Shi‐Gang Sun**: resources, project administration. **Maolin Yang**: conceptualization, software, investigation. **Chunjing Hu**: data curation, formal analysis. **Kang Zhang**: data curation, formal analysis. **Yuan Tian**: data curation. **Bixian Ying**: conceptualization, data curation, investigation. **Chao Li**: formal analysis, software. **Yinguo Xiao**: software, formal analysis, data curation. **Guifan Zeng**: data curation, formal analysis. **Jiyuan Xue**: data curation. **Peng Zhang**: data curation, formal analysis. **Wei Li**: data curation, formal analysis. **Huaqiang Chen**: software, formal analysis. **Chunpu Li**: methodology, investigation. **Peter Nagel**: data curation, formal analysis, software. **Fanghua Ning**: supervision, data curation, formal analysis. **Tao Zeng**: software, data curation, formal analysis. **Jin Yi**: software, formal analysis. **Tian Qiu**: software, formal analysis. **Qingsong Wang**: conceptualization, data curation, investigation, writing – review and editing. **Changming Qu**: resources, methodology. **Stefan Schuppler**: data curation, software, formal analysis. **Jia‐Wei Wang**: conceptualization, supervision.

## Conflicts of Interest

The authors declare no conflicts of interest.

## Supporting information




**Supporting File 1**: anie74206‐sup‐0001‐SuppMat.docx.

## Data Availability

The data that support the findings of this study are available on request from the corresponding author. The data are not publicly available due to privacy or ethical restrictions.
