# Supplementary material for: Reinforcing Particle Architectural Stability of Li‐Rich Cathode With Enhanced Anionic Redox Reaction Reversibility
Source: Angew Chem Int Ed Engl. 2026 Aug 14;65(41):e6490997. doi: 10.1002/anie.6490997 (PMC13638103; doi:10.1002/anie.6490997)
Supplement: Supplementary file 1 [file ANIE-65-e6490997-s001.docx]

Supporting Information
©Wiley-VCH 2021
69451 Weinheim, Germany

**Reinforcing Particle Architectural Stability of Li-Rich Cathode with Enhanced Anionic Redox Reaction Reversibility**

Yizhen Huang, Bixian Ying, Chunpu Li, Maolin Yang,* Kang Zhang, Guifan Zeng, Jiyuan Xue, Yuan Tian, Wei Li, Chunjing Hu, Tao Zeng, Peng Zhang, Chao Li, Yinguo Xiao, Huaqiang Chen, Tian Qiu, Stefan Schuppler, Peter Nagel, Jin Yi, Changming Qu, Jia-Wei Wang,* Fanghua Ning,* Qingsong Wang,* Yu Qiao,* and Shi-Gang Sun

Experimental Procedures

**Material Preparation**

**(a) Preparation of the Li_1.13_Ni_0.30_Mn_0.57_O_2_ (LRLO)**

The hydroxide precursor of Ni_0.35_Mn_0.65_(OH)_2_ was prepared via a co-precipitation route. NiSO_4_·6H_2_O and MnSO_4_·5H_2_O were dissolved in deionized water molar ratio of 0.35:0.65 to obtain a 2 mol L^-1^ aqueous solution. This solution, together with a 2.0 mol L^-1^ NaOH solution and a 0.5 mol L^-1^ NH_3_∙H_2_O solution, were simultaneously fed into a 5 L reactor under continuous stirring. The reaction was conducted at 55 °C, while the pH was maintained at ~8.0 under a N_2_ atmosphere throughout the co-precipitation process. The resulting microspherical (MnNi)(OH)_2_ precursor was collected by filtration, thoroughly washed with deionized water, and dried at 80 °C.

The Ni_0.35_Mn_0.65_(OH)_2_ precursor was thoroughly homogenized with a stoichiometric amount of LiOH·H_2_O (with a 10% excess). The resulting mixture was first preheated at 500 °C for 5 h in the muffle furnace, followed by calcination at 900 °C for 15 h in air. After natural cooling to room temperature, the Li_1.13_Ni_0.30_Mn_0.57_O_2_ (LR) were obtained.

**(b) Synthesis of the LR-F**

The Ni_0.35_Mn_0.65_(OH)_2_ precursor was thoroughly homogenized with a stoichiometric amount of LiOH·H_2_O (with a 10% excess) and LiF. The resulting mixture was first preheated at 500 °C for 5 h in the muffle furnace, followed by calcination at 900 °C for 15 h in air. After natural cooling to room temperature, the LR-F were obtained.

**Electrode preparation**

To prepare the LRLO cathode electrode, the active materials LRLO (80 wt%) with carbon black (Super P, Canrd, 10 wt%), and polyvinylidene fluoride (PVDF, Canrd, 10 wt%) in N-methyl pyrrolidone (NMP, J&K.) were mixed to form a homogenous slurry. The slurry was spread onto a carbon coated aluminum current collector (Suzhou Sinero Technology Co., Ltd.) and dried at 80 °C for 12 hours in muffle furnace (MSK, KSL-1100X-S). The electrode was pressed and cut into circular slices with the active material loading of about 4 mg cm^-2^.

**Cell assembly and electrochemical measurements**

The cathode electrode pieces were further dried at 110 °C under vacuumed overnight before quickly transferred to a glove box filled with Ar (O_2_ < 0.01 ppm and H_2_O < 0.01 ppm). In this paper, all LRLO || Li half cells were assembled using CR2032 type coin cells, Li foil as anode (thickness, 0.5 mm), a porous polypropylene film (Celgard 2300) as separator, and 1.0 M LiPF_6_ in ethylene carbonate (EC): dimethyl carbonate (EMC) (3:7 v/v, Do Do Chem) as electrolyte. The electrochemical performance test of LRLO cathode materials was carried out through constant-current constant-voltage charging and constant-current discharging protocols on a Neware battery test system (CT-4008T-5 V 10 mA) at 26 °C. Initially, the battery was charged at a constant current density of 10 mA g-1 (0.05C, 1C = 200 mA g^-1^) to 4.8 V. The initial discharge occurred at a constant current density of 10 mA g^-1^ (0.05C), with the lower cut-off voltage of 2 V. During the subsequent cycling, the cells were charged at a constant current density of 100 mA g^-1^ (0.5C) up to 4.8 V and then charged at a constant voltage of 4.8 V until it reached a cutoff current density of 10 mA g^-1^ (0.05C). The discharge was carried out at a fixed current density of 100 mA g^-1^ (0.5C), with the voltage cut-off of 2.0 V. The Li^+^ chemical diffusion coefficients (D_Li_^+^) were determined using the GITT, with charging/discharging at 0.1C for 1 hour, followed by relaxation for 8 hours between 2.0 V and 4.8 V. The following simplified formula was used to calculate the Li^+^ diffusion coefficient: ^[1]^

$D_{Li^{+}}=\frac{4}{\pi\tau}\left. \left( \frac{mV_{M}}{MS} \right. \right)^{2}\left. \left( \frac{{\Delta E}_{S}}{{\Delta E}_{\tau}} \right. \right)^{2}$	(1)

where *D*_Li_^+^ (cm^2^ s^-1^) is the lithium-ion diffusion coefficient, *τ* (s) is the relaxation time, *S* (cm^2^) is the electrode/electrolyte interface area, *V*_m_ (cm^3^ mol^-1^) is the molar volume, *M*_B_ (g mol^-1^) is the molar mass of the electrode material, *m*_B_ (g) is the electrode active-material mass, Δ*E*_s_ is the voltage difference between the steady-state potentials before and after the application of current pulse, and Δ*E*_τ_ is the voltage difference between the cell potential at the start and the end of the current pulse.

Cyclic voltammetry (CV) measurements were carried out on a CHI660C electrochemical workstation within a potential window of 2.0–4.8 V, at scan rates ranging from 0.1 to 1.0 mV s-1. The Li^+^ diffusion coefficients were determined from the linear relationship between the peak current (*I_p_*) and the square root of the scan rate (*v*^1/2^). According to the Randles-Sevcik equation:

$I_{p}=(2.69\times{10}^{5})n^{3/2}A{D_{Li}}^{1/2}C_{Li}v^{1/2}$	(2)

where *I_p_* represents the peak current, n is the number of electrons transferred in the redox reaction, A is the electrode area, *D*_Li_ is the Li^+^ diffusion coefficient within the electrode material, v is the scan rate, and *C*_Li_ is the change in Li^+^ concentration before and after the reaction.

According to the Randles-Sevcik equation, in a diffusion-controlled electrochemical process, the redox peak current (*I_p_*) exhibits a linear relationship with the square root of the scan rate (*v*^1/2^). According, the Li^+^ diffusion coefficient (*D*_Li_) is proportional to the slope of the linear relationship between Ip and *v*^1/2^. Therefore, the slope obtained from the linear fitting of Ip versus v1/2 can be used to qualitatively compare Li^+^ diffusion kinetics, where a larger slope indicates faster ion transport^[2]^.

Electrochemical impedance spectroscopy analysis (EIS, 100 kHz~0.01 Hz) were performed on a biological multichannel electrochemical workstation (VSP300).

In the DRT spectra, the relaxation time (τ) can be associated with specific electrochemical processes. Following established DRT interpretation procedures (such as the framework proposed by Francesco Ciucci and co-workers), the DRT peaks were assigned and integrated to quantify the corresponding resistance contributions ^[3-4]^.

**Characterizations**

**Hard X-ray absorption spectroscopy (hXAS) measurement**

Hard X-ray absorption spectroscopy (hXAS) measurements at Ni and Mn K-edge were performed at BL11B of the Shanghai Synchrotron Radiation Facility (SSRF). All the samples were washed three times with DMC, dried in the vacuum antechamber of an argon-filled glovebox, and then the samples were sealed with Kapton film to avoid exposure to air. The data were measured under both fluorescence mode and transmission mode. The reference was measured under the transmission mode. Data reduction and analysis were carried out using the Demeter program pack with Athena.

**Synchrotron-based X-ray diffraction (sXRD) measurement**

Synchrotron powder XRD patterns with a wavelength of λ= 0.6887 Å were collected at the BL14W1 beamline of the Shanghai Synchrotron Radiation Facility. Rietveld refinements were conducted using the GSAS-II Suite.

**Neutron powder diffraction (NPD)**

The neutron powder diffraction experiments were performed on a time-of-flight high-resolution neutron diffractometer at the China Spallation Neutron Source. Each sample was loaded into a vanadium nickel (VNi) can, and the diffraction pattern was collected over a wavelength range of 0.1-3.9 Å at room temperature under vacuum. The Rietveld refinement was carried out to analyze the crystal structures using GSAS-II Suite..^[5]^

**Resonant inelastic X-ray scattering (RIXS)**

Resonant inelastic X-ray scattering (RIXS) experiments were conducted at beamline U41-PEAXIS at BESSY II, located at Helmholtz-Zentrum Berlin (HZB). A vacuum suitcase was used to transfer the sample from an N_2_-filled glovebox to the end station chamber. The spectrometer was positioned at specular conditions relative to 60° scattering angle and was optimized to a combined resolution of 90 meV using a carbon tape. The O K-edge RIXS spectra of the samples were collected at an excitation energy of 531.0 eV. The acquisition time for each pattern was 30 min.^[6]^

**Scanning electron microscopy (SEM)**

The morphologies and elemental proportions of active materials were obtained by scanning electron microscopy (SEM, ZEISS Gemini SEM 500).

**Soft X-ray absorption spectroscopy (sXAS) measurement**

The electrodes were cycled to the target voltages. Subsequently, the cells were disassembled in the glovebox, and the electrodes were thoroughly washed with dimethyl carbonate (DMC) to remove electrolyte residues. They were then mounted onto the sample plate and transferred to the beamline using a transfer case to mitigate air contamination. sXAS data were performed at IQMT's soft X-ray beamline WERA at the Karlsruhe Light Source, Germany. sXAS measurements at O K were carried out simultaneously in fluorescence yield (FY) and total electron yield (TEY) detection mode. A resolution of 0.19 eV at the O K edge was used. A NiO reference was used for data energy calibration.^[7-8]^.

The sXAS spectra were recorded over a broad photon-energy range from 500 to 605 eV, covering regions well below and well above the absorption features of the samples. The FY signal was normalized following an established procedure:

(1) I₀ normalization: The raw FY signals collected from four fluorescence detectors positioned at different angles were summed. The incident X-ray intensity, I₀, was measured using the drain current from a freshly Au-coated mesh inserted into the beam path upstream of the sample, before the X-rays impinged on the sample. Dark counts, corresponding to the residual signal recorded with the shutter closed, were subtracted. The normalized FY signal was then obtained by dividing the summed raw FY signal by the corrected incident intensity.

(2) Background subtraction: A linear sloping background was removed by fitting a straight line to the flat low-energy region of the sXAS spectrum, from 500 to 522 eV, where no absorption features are present.

(3) Edge-jump normalization: The spectrum was normalized by setting the flat low-energy pre-edge region to zero and the post-edge region to unity, corresponding to a unit edge jump. The photon-energy range selected for the post-edge normalization was 590–605 eV, which lies well above the absorption features. The normalization is not sensitive to the exact choice of this range, as it is sufficiently far above any compound-specific resonances.

**Integrated focused ion beam scanning electron microscope (FIB-SEM)/High-resolution transmission electron microscopy (HR-TEM)**

Cross-sectional sample preparation: the cross-sectional lamella specimens were cut by using ZEISS Cross Beam 350 FIB-SEM system, which were then milled down by using Ga-ion operated at a voltage reducing from 30 to 2 kV and the ionic beam current reducing from 3 nA to 10 pA.

The morphologies of active materials were obtained by high-resolution transmission electron microscopy (HR-TEM, JEOL JEM-F200).

**Solid-state ^7^Li NMR spectroscopy measurements**

The electrode material was scraped from the Al foil and loaded into a 1.9 mm magic angle spinning (MAS) NMR rotor. ^7^Li solid-state NMR experiments were conducted using a 400 MHz Bruker ADVANCE-III spectrometer equipped with a triple-resonance 1.9 mm MAS probe. The ^7^Li Larmor frequency of 155.51 MHz was used. The MAS frequency was 30 kHz, and the probe temperature was maintained at 323 K. The transmitter offset was set to 1000 ppm. The ^7^Li MAS NMR spectra were acquired using the pj-MATPASS (pj = projection, MAT: magic-angle turning, PASS: phase-adjusted sideband separation) pulse sequence. The π/2 pulse length was 1.6 μs, and the recycle interval was 20 ms. The ^7^Li shifts were calibrated with respect to a 1 M LiCl solution (0 ppm).

**Integrated FIB-SEM/Time of flight secondary ion mass spectroscopy (TOF-SIMS)**

Cross-sectional sample preparation: the cross-sectional lamella specimens were cut by using ZEISS Cross Beam 350 FIB-SEM system, which were then milled down by using Ga-ion operated at a voltage reducing from 30 to 2 kV and the ionic beam current reducing from 3 nA to 10 pA.

TOF-SIMS acquisition: Measurements were conducted using a 30 keV, 100 pA primary Bi_3_⁺ ion beam, acquiring 1024 × 1024 pixels elemental maps (7 × 7 to 20 × 20 μm fields of view) with lateral resolutions of 20-80 nm/pixel. Data processing: Twenty sequential frames were collected to achieve ~100 nm depth profiling. The resultant 4D data cube (x, y, z, m/z) enabled extraction of specific ion intensity distributions through post-acquisition mass-resolved slicing.

Small-angle X-ray scattering (SAXS)

Synchrotron scattering experiments were performed at the Shanghai Synchrotron Radiation Facility (SSRF) beamline BL16B1. The incident X-ray wavelength was 0.124 nm, with a photon flux of approximately 3.9 × 10¹¹ phs/s and an energy of 10 keV. All sample signals were acquired using a Pilatus 2M detector. The sample‑to‑detector distance was calibrated to be 0.6 m using a Silver Behenate (AgBh) standard.

**Accelerating Rate Calorimetry (ARC)**

ARC tests were conducted by placing the pouch cell in an adiabatic chamber with an initial temperature of 50 °C, a heating step of 5 °C, and a self-heating rate (SHR) threshold of 0.02 °C·min^-1^.

**Mechanical Property Testing**

The electrically conductive of samples was measured by Kelvin four-wire combining dual-disk electrodes measurement (PRCD1100, IEST).

$D=\frac{m}{S L}$	(3)

where *D* (g cm^-3^) denotes the compacted density; m denotes the sample mass; S denotes the cross-sectional area of the sample; and L denotes the sample thickness.

The compaction density of samples is measured by a high-precision servo motor and displacement sensor with an adaptive pressure regulation control system based on the multiple measurement modes (PRCD1100, IEST).

$R=\rho\frac{L}{S}$	(4)

$\sigma_{e}=\frac{L}{R S}$	(5)

where *R* (Ω) denotes resistance; *ρ* (Ω cm) denotes resistivity; L denotes the sample thickness; S denotes the cross-sectional area; and σ_e_ (μs cm^-1^) denotes the conductivity.

The force performance of single particle was measured by a high-precision nano-control force and displacement sensors based on a single particle force tester (IEST, SPFT-2000).

$P=A \frac{F_{yk}}{\pi d^{2}}$	(6)

where *P* (MPa) denotes hardness; A denotes a calculation coefficient, taken as 2.48 or 2.8; *F_yk_* (mN) denotes the crushing force; and d (μm) denotes the particle diameter.

Ion chromatography (IC)

The fluorine concentrations of the samples were quantitatively determined using an ion chromatography (IC) system (Thermo Fisher, ICS-1100) equipped with an anion-exchange column and a suppressed conductivity detector. The eluent consisted of a mixture of 4.5 mM Na_2_CO_3_ and 1.4 mM NaHCO_3_ with a flow rate of 1.2 mL min^-1^. To distinguish between surface residual fluorine and lattice-incorporated fluorine, three sample preparation procedures were systematically performed:

(1) Total fluorine content determination: To quantify the overall fluorine concentration, a specific amount of powder sample was subjected to a standard alkali fusion process. The powder was mixed with NaOH in a nickel crucible and heated at 600 °C for 2 h to ensure complete decomposition. The resulting fused product was dissolved in deionized water, neutralized with a dilute acid solution, transferred to a 50 mL volumetric flask, and diluted to volume. The solution was subsequently filtered through a 0.22 μm polytetrafluoroethylene (PTFE) membrane filter prior to IC analysis.

(2) Surface residual fluorine extraction: To isolate water-soluble or weakly bound fluorine species present on the material surface (e.g., residual LiF or surface species), a predetermined amount of powder sample was dispersed in deionized water. The suspension was sonicated at room temperature for 30 min to promote sufficient leaching. The mixture was then centrifuged at 8000 rpm for 10 min. The supernatant was collected, diluted to a fixed volume, filtered, and analyzed by IC to determine the surface residual F content.

(3) Lattice-incorporated fluorine quantification: The solid residue obtained after the mild washing/leaching process (Step 2) was thoroughly rinsed with deionized water three times and dried under vacuum at 80 °C overnight. The dried residue was subsequently subjected to the same alkali fusion and dissolution procedures described in Protocol (1). The resulting solution was analyzed by IC to quantify the fluorine content retained within the crystal structure.

Density Functional Theory (DFT) Calculations

Density functional theory (DFT) calculations were carried out using the projected augmented wave (PAW) approach implemented in the DS-PAW package within Device Studio^[9-10]^. Spin polarization was considered. The generalized gradient approximation (GGA) with the Perdew-Burke-Ernzerhof (PBE) exchange-correlation functional was employed^[11]^. A plane-wave basis set with a cutoff energy of 520 eV was adopted to expand the Kohn–Sham wavefunctions^[12]^. Brillouin zone integration was performed on a 2 × 2 × 1 Monkhorst-Pack k-point mesh^[13]^. To describe the strong on-site Coulomb interaction of 3d electrons, the GGA+U scheme^[14]^ was applied, with effective U values of 5 eV for Ni and 4 eV for Mn. The electronic self-consistent field iterations were converged to 10^-5^ eV. The forces on all atoms in ionic relaxations were converged to 0.05 eV Å^-1^. The structural model of the LR material contains 240 atoms, corresponding to the stoichiometric formula of Li_1.13_Ni_0.30_Mn_0.57_O_2_. The highly delithiated LR material was constructed by extracting 60 Li ions from the pristine structure. The F-doped system was obtained by replacing two oxygen sites within the 120 lattices oxygen atoms with fluorine ions.

Quantitative Analysis of (010) Facet Exposure via March-Dollase Preferred Orientation Model

To quantitatively evaluate the morphological evolution and the relative exposure of the electrochemically active (010) facets, we incorporated the March–Dollase preferred-orientation model into the Rietveld refinement of the XRD patterns.

The March–Dollase model, a well-established mathematical model in crystallograph, accounts for preferred-orientation effects by correcting the theoretical diffraction intensity through a pole-density distribution function^[15]^:

$P=\left( r^{2}{cos}^{2}\alpha+r^{-1}{sin}^{2}\alpha\right)^{-3/2}$	(7)

Here, P is the orientation correction factor, α is the spatial angle between the normal of the Selected diffraction plane and the sample reference axis, and r is the refinable March parameter quantifying the morphological anisotropy.

For the specific (010) diffraction peak, the angle α with respect to the [010] preferred axis is exactly 0°. Consequently, the correction formula simplifies directly to:

$P=r^{-3}$	(8)

This simplified equation allows us to quantitatively calculate the exact degree of suppression or amplification of the (010) facets.

Results and Discussion

**Figure S1. Optimized surface structure models used in first-principles calculations (side view).** (a) Clean (001) surface, (b) F-incorporated (001) surface, (c) clean (010) surface, (d) F-incorporated (010) surface.

**Figure S2. Optimized surface structure models used in first-principles calculations (top view).** (a) Clean (001) surface, (b) F-incorporated (001) surface, (c) clean (010) surface, (d) F-incorporated (010) surface.

**Figure S3**. The SEM image of (a) LR and (b) LR-F cathodes.

**Figure S4**. SEM images of (a) LR-F-0.5 and (b) LR-F-2 cathodes, and the corresponding cross-sectional morphologies of (c) LR-F-0.5 and (d) LR-F-2 cathodes.

**Figure S5. sXRD patterns and refined results of LR and LR-F powder.**

**Figure S6**. Discharge curves at diﬀerent rates of LR and LR-F cathodes at 45 °C, 2.0-4.8 V.

**Figure S7.** (a) Cyclic voltammetry curves recorded at scan rates ranging from 0.1 to 1.0 mV s^-1^ for (a) LR and (b) LR-F cathodes; (c) linear fitting of peak current (*I_p_*) versus the square root of scan rate (v^1/2^) for both charge and discharge processes of LR and LR-F cathodes.

**Figure S8.** Electrochemical impedance spectroscopy (EIS) analysis of LR and LR-F cathodes after 30 cycles. (a) Nyquist plots with real (Z′) and imaginary (Z″) impedance components. (b) Distribution of relaxation time (DRT) spectra, γ(τ), as a function of relaxation time (τ). The green shaded region represents charge transfer resistance (*R_ct_*), and the pink shaded region corresponds to solid-state resistance (*R_d_*).

**Figure S9**. Powder resistivity & compaction density measurement system (PRCD1100, IEST).

**Figure S10**. Single particle force tester (IEST, SPFT-2000).

**Figure S11**. (a) Normalized sXAS spectra of LR-F and LR (OCV, 4.8 V) in the 523–538 eV range. (b) Same spectra over 500–605 eV, with a zoom of the 590–605 eV region.

**Figure S12**. Electrochemical charging curves online with OEMS detection of LR and LR-F cathodes (0.2C rate, OCV-4.8 V vs. Li⁺/Li).

**Figure S13**. Crystal structure of (a) Li_1.13_Ni_0.30_Mn_0.57_O_2_ (pristine), (b) Li_1.13_Ni_0.30_Mn_0.57_O_2_-F (pristine), (c) Li_0.08_Ni_0.30_Mn_0.57_O_2_ (delithiated) and (d) Li_0.08_Ni_0.30_Mn_0.57_O_2_-F (delithiated).

**Figure S14**. The oxygen-projected density of states was calculated for LR and LR-F at pristine state.

**Figure S15**. (a) Crystal structure model of the LR-F cathode, with the selected TM-layer plane highlighted for electronic structure analysis. Top-view atomic configurations of the transition metal layer for both the undoped (b) LR and (c) LR-F cathodes in an identical highly delithiated state, the white dashed triangles and circles indicate the specific local environments around the O and F sites, respectively. The corresponding charge density distribution maps of (d) LR and (e) LR-F cathodes.

**Figure S16**. The GITT potential profiles of LR and LR-F electrodes during the initial cycle (0.1C rate, 4.8-2.0 V vs. Li⁺/Li).

**Figure S17.** Average discharge voltage of LR and LR-F electrodes over the 1–300 cycle range.

**Figure S18.** (a) Initial charge–discharge curves of LR-F-0.5 and LR-F-2 cathodes measured at 25 °C, 0.05C, and 2.0-4.8 V. (b) Cycle performance of LR-F-0.5 and LR-F-2 cathodes measured at 25 °C, 0.5C, 2.0-4.8 V.

**Figure S19.** *k^3^*-weighted Fourier transform magnitudes of the Mn K-edge with EXAFS best-fit analysis for LR and LR-F cathodes at fully charged state (4.8 V) after 100 cycles.

**Figure S20.** TOF-SIMS maps of the normalized elemental distribution of F in (a) LR and (b) LR-F powder.

**Figure S21.** (a) Charge-discharge curves after 50 cycles at 25 °C, 0.5C, 2.0-4.8 V. Raw ion intensity curves of TOF-SIMS characterization of the (b)LR and (c)LR-F cathodes after 50 cycles, 25 °C, 0.5C, and 2.0–4.8 V.

**Figure S22.** Discharge median voltage evolution of (a) the Gr||LR-F single-layer pouch-type full-cell (0.5 Ah, 2.5-4.75 V) and (b) the Gr||LR-F multi-layer pouch-type full-cell (2.0 Ah, 2.5-4.65 V). Charge–discharge curves at selected cycles of (c) the Gr||LR-F single-layer pouch-type full-cell and (d) the Gr||LR-F multi-layer pouch-type full-cell.

**Table S1**. Rietveld refinement of LR and LR-F (pristine powder) sXRD.

| Li_1.13_N_i0.30_Mn_0.57_O_2_ (*R*$\bar{\text{3}}$*m*) | | | | | | | | | | |
| --- | --- | --- | --- | --- | --- | --- | --- | --- | --- | --- |
| Atomic  Occupancies | Wyckoff | Atom | X | | | | Y | | | Z |
|  | 3*b* | Li1 | 0 | | | | 0 | | | 0.5 |
|  | 3*a* | Li2 | 0 | | | | 0 | | | 0 |
|  | 3*b* | Ni1 | 0 | | | | 0 | | | 0.5 |
|  | 3*a* | Ni2 | 0 | | | | 0 | | | 0 |
|  | 3*a* | Mn | 0 | | | | 0 | | | 0 |
|  | 6*c* | O1 | 0 | | | | 0 | | | 0.2668 |
| Lattice  Parameters | *a*/Å | | | *b*/Å | | | | *c*/Å | | |
|  | 2.8729 | | | 2.8729 | | | | 14.2784 | | |
| *r* | 1.104 | | | | | | | | | |
| Li_1.13_N_i0.30_Mn_0.57_O_1.993_F_0.007_ (*R*$\bar{\text{3}}$*m*) | | | | | | | | | | |
| Atomic  Occupancies | Wyckoff | Atom | | X | | Y | | | Z | |
|  | 3*b* | Li1 | | 0 | | 0 | | | 0.5 | |
|  | 3*a* | Li2 | | 0 | | 0 | | | 0 | |
|  | 3*b* | Ni1 | | 0 | | 0 | | | 0.5 | |
|  | 3*a* | Ni2 | | 0 | | 0 | | | 0 | |
|  | 3*a* | Mn | | 0 | | 0 | | | 0 | |
|  | 6*c* | O1 | | 0 | | 0 | | | 0.26212 | |
|  | 6*c* | F1 | | 0 | | 0 | | | 0.26212 | |
| Lattice  Parameters | *a*/Å | | | | *b*/Å | | | *c*/Å | | |
|  | 2.8743 | | | | 2.8743 | | | 14.2909 | | |
| *r* | 0.861 | | | | | | | | | |

**Table S2**. Compaction density and electrical conductivity of LR and LR-F samples under different pressures.

| Pressure (Mpa) | Compaction density  (g cm^-3^) | | Conductivity  (μs cm^-1^) | |
| --- | --- | --- | --- | --- |
|  | LR | LR-F | LR | LR-F |
| 10 | 2.06 | 2.36 | 4.70 | 9.90 |
| 30 | 2.15 | 2.41 | 10.8 | 18.5 |
| 50 | 2.20 | 2.44 | 14.8 | 24.0 |
| 70 | 2.25 | 2.46 | 18.2 | 28.4 |
| 90 | 2.31 | 2.49 | 21.3 | 32.3 |
| 110 | 2.38 | 2.52 | 24.2 | 36.2 |
| 130 | 2.44 | 2.56 | 27.1 | 40.0 |
| 150 | 2.51 | 2.60 | 29.8 | 43.9 |
| 170 | 2.58 | 2.64 | 32.5 | 47.7 |
| 190 | 2.64 | 2.68 | 35.0 | 51.5 |
| 210 | 2.69 | 2.72 | 37.3 | 55.0 |
| 230 | 2.74 | 2.76 | 39.4 | 58.5 |
| 250 | 2.78 | 2.80 | 41.4 | 61.8 |
| 270 | 2.83 | 2.84 | 43.3 | 65.1 |
| 290 | 2.87 | 2.87 | 45.1 | 68.3 |
| 310 | 2.90 | 2.91 | 46.8 | 71.3 |
| 330 | 2.94 | 2.94 | 48.5 | 74.3 |
| 350 | 2.97 | 2.98 | 50.0 | 77.1 |

**Table S3**. The test results of the mechanical performance of a single particle of LR sample.

| Particle number | crushing force (mN) | Pressure (MPa) |
| --- | --- | --- |
| 1 | 2.9694 | 50.0595 |
| 2 | 3.4986 | 69.5849 |
| 3 | 3.283 | 69.6487 |
| 4 | 3.4398 | 75.28 |
| 5 | 2.8224 | 57.9612 |
| 6 | 2.45 | 50.3135 |
| 7 | 2.7048 | 55.5461 |
| 8 | 3.3222 | 64.0277 |
| average value | 3.0613 | 61.5527 |

**Table S4.** The test results of the mechanical performance of a single particle of LR-F sample.

| Particle number | crushing force (mN) | Pressure (MPa) |
| --- | --- | --- |
| 1 | 12.544 | 122.251 |
| 2 | 10.9172 | 106.397 |
| 3 | 13.7396 | 133.903 |
| 4 | 9.555 | 89.1162 |
| 5 | 10.2214 | 99.6156 |
| 6 | 12.8282 | 114.607 |
| 7 | 12.5832 | 107.783 |
| 8 | 13.7592 | 122.925 |
| average value | 12.0185 | 112.0745 |

**Table S5.** Comparison of fabrication parameters for LR and LR-F cathodes in coin-cell assembly.

| Comparison project | LR | LR-F |
| --- | --- | --- |
| Areal mass loading (mg cm^-2^) | 6.08 | 7.13 |
| calendaring pressure  (rolling clearance, mm) | 1.3 | 1.3 |
| electrode Thickness (μm) | 34.0 | 33.5 |
| tap density (g cm^-3^) | 1.79 | 2.14 |
| active material content (wt.%) | 80 | 80 |

**Table S6.** Crystallographic parameters of the LR-F refined by the Rietveld refinement of NPD.

| Li_1.13_Ni_0.30_Mn_0.57_O_1.993_F_0.007_ | | | | | | | | |
| --- | --- | --- | --- | --- | --- | --- | --- | --- |
| Atomic  Occupancies | Wyckoff | Atom | X | | Y | Z | | Occ. |
|  | 3*b* | Li1 | 0 | | 0 | 0.5 | | 0.9895 |
|  | 3*a* | Li2 | 0 | | 0 | 0 | | 0.1405 |
|  | 3*b* | Ni1 | 0 | | 0 | 0.5 | | 0.0105 |
|  | 3*a* | Ni2 | 0 | | 0 | 0 | | 0.2895 |
|  | 3*a* | Mn | 0 | | 0 | 0 | | 0.5700 |
|  | 6*c* | O1 | 0 | | 0 | 0.25817 | | 0.9964 |
|  | 6*c* | F1 | 0 | | 0 | 0.25817 | | 0.0036 |
| Lattice  Parameters | *a*/Å  2.8726 | | | *b*/Å  2.8726 | | | *c*/Å  14.2691 | |

**Table S7.** Ion Chromatography (IC) results of LR-F sample.

| Nominal composition | Theoretical F content (mol%) | Fluorine distribution state | Actual Fluorine content (mol%) |
| --- | --- | --- | --- |
| Li_1.13_Ni_0.30_Mn_0.57_O_1.99_F_0.01_ | 1 | Total fluorine | 0.75 |
|  |  | Surface-residual fluorine | 0.60 |
|  |  | Lattice-incorporated fluorine | 0. 13 |

**Table S8.** Comparison of average discharge voltage decay rates (mV cycle^-1^) of LR and LR-F samples during the 1–300 cycle range

| Cycling stage | LR | LR-F |
| --- | --- | --- |
| 1-300 cycles | 1.66 mV cycle^-1^ | 0.70 mV cycle^-1^ |

**Table S9:** Relationship between fluorine content, particle morphology, internal compactness, and electrochemical performance.

| sample | primary-particle morphology | Internal compactness of secondary particles | electrochemical performance | |
| --- | --- | --- | --- | --- |
|  |  |  | First-cycle Coulombic efficiency (%) | Capacity retention after 200th cycles (%) |
| LR | distinctly plate-like | low | 78.1 | 82.9 |
| LR-F-0.5  (0.5 mol%) | plate-like | high | 82.1 | 88.6 |
| LR-F  (1mol%) | distinctly equiaxed | superior | 84.9 | 96.7 |
| LR-F-2 (2 mol%) | distinctly equiaxed | high | 83.4 | 92.0 |

**Table S10.** Rietveld refinement of LR (after 200 cycles) sXRD.

| Li_1.13_N_i0.30_Mn_0.57_O_2_ (*R*$\bar{\text{3}}$*m*) Wt. fraction **95.2%** | | | | | | | | | | |
| --- | --- | --- | --- | --- | --- | --- | --- | --- | --- | --- |
| Atomic  Occupancies | Wyckoff | Atom | X | | Y | | Z | | | Occ. |
|  | 3*b* | Li1 | 0 | | 0 | | 0.5 | | | 0.9780 |
|  | 3*a* | Li2 | 0 | | 0 | | 0 | | | 0.1300 |
|  | 3*b* | Ni1 | 0 | | 0 | | 0.5 | | | 0.0220 |
|  | 3*a* | Ni2 | 0 | | 0 | | 0 | | | 0.3000 |
|  | 3*a* | Mn | 0 | | 0 | | 0 | | | 0.56 |
|  | 6*c* | O1 | 0 | | 0 | | 0.2668 | | | 1 |
| Lattice  Parameters | *a*/Å | | *b*/Å | | | | | *c*/Å | | |
|  | 2.87201 | | 2.8721 | | | | | 14.3465 | | |
| LiNi_0.5_Mn_1.5_O_4_ (*Fd-*$\bar{\text{3}}$*m*) Wt. fraction **4.8%** | | | | | | | | | | |
| Atomic  Occupancies | Wyckoff | Atom | X | Y | | Z | | | Occ. | |
|  | 32*e* | O | 0.2048 | 0.2048 | | 0.2048 | | | 1 | |
|  | 16*d* | Mn | 0.5 | 0.5 | | 0.5 | | | 0.75 | |
|  | 16*d* | Ni | 0.5 | 0.5 | | 0.5 | | | 0.25 | |
|  | 8*a* | Li | 0.1250 | 0.1250 | | 0.1250 | | | 1 | |
| Lattice  Parameters | *a*/Å | | | | | | | | | |
|  | 8.1455 | | | | | | | | | |

**Table S11.** Rietveld refinement of LR-F (after 200 cycles) sXRD.

| Li_1.13_N_i0.30_Mn_0.57_O_2_ (*R*$\bar{\text{3}}$*m*) | | | | | | | | |
| --- | --- | --- | --- | --- | --- | --- | --- | --- |
| Atomic  Occupancies | Wyckoff | Atom | | X | Y | | Z | Occ. |
|  | 3*b* | Li1 | | 0 | 0 | | 0.5 | 0.9854 |
|  | 3*a* | Li2 | | 0 | 0 | | 0 | 0.1446 |
|  | 3*b* | Ni1 | | 0 | 0 | | 0.5 | 0.0146 |
|  | 3*a* | Ni2 | | 0 | 0 | | 0 | 0.2854 |
|  | 3*a* | Mn | | 0 | 0 | | 0 | 0.5700 |
|  | 6*c* | O1 | | 0 | 0 | | 0.2639 | 1 |
| Lattice  Parameters | *a*/Å | | *b*/Å | | | *c*/Å | | |
|  | 2.8762 | | 2.8762 | | | 14.3828 | | |

**Table S12.** Key electrode parameters for pouch-cell assembly.

| **Parameter** | **LR-F** |
| --- | --- |
| active material content (wt.%) | 93 |
| calendaring Pressure (kN) | 150 |
| tap density (g cm^-3^) | 0.013 |
| electrode Thickness (μm) | 100 |
| areal mass loading (g cm^-2^) | 1.2 |

# Reference

[1]	S.-T. Myung, S. Komaba, K. Hosoya, N. Hirosaki, Y. Miura, N. Kumagai, *Chemistry of Materials* **2005**, *17*, 2427-2435.

[2]	J. Sun, H. Yang, J. Shen, H. Qi, M. Sun, Y. Lou, Y. Yao, X. Rui, Y. Shao, X. Wu, Y. Yu, *Energy Environ. Sci.* **2025**, *18*, 4335-4347. <http://doi.org/10.1039/d5ee00430f>

[3]	Z. Wang, Y. Wang, B. Py, A. Maradesa, J. Liu, T. H. Wan, M. Saccoccio, F. Ciucci, *ACS Electrochem.* **2025**, *1*, 2680-2689. <http://doi.org/10.1021/acselectrochem.5c00334>

[4]	Y. Lu, C.-Z. Zhao, J.-Q. Huang, Q. Zhang, *Joule* **2022**, *6*, 1172-1198. <http://doi.org/10.1016/j.joule.2022.05.005>

[5]	B. H. Toby, R. B. Von Dreele, *J. Appl. Crystallogr.* **2013**, *46*, 544-549. <http://doi.org/10.1107/s0021889813003531>

[6]	C. Schulz, K. Lieutenant, J. Xiao, T. Hofmann, D. Wong, K. Habicht, *J. Synchrotron Radiat.* **2020**, *27*, 238-249. <http://doi.org/10.1107/S1600577519014887>

[7]	M. Merz, D. Fuchs, A. Assmann, S. Uebe, H. v. Löhneysen, P. Nagel, S. Schuppler, *PRB* **2011**, *84*, 014436. <http://doi.org/10.1103/PhysRevB.84.014436>

[8]	M. Merz, P. Nagel, C. Pinta, A. Samartsev, H. v. Löhneysen, M. Wissinger, S. Uebe, A. Assmann, D. Fuchs, S. Schuppler, *PRB* **2010**, *82*, 174416. <http://doi.org/10.1103/PhysRevB.82.174416>

[9]	P. E. Blochl, *Phys Rev B Condens Matter* **1994**, *50*, 17953-17979. <http://doi.org/10.1103/physrevb.50.17953>

[10]	D. J. G. Kresse, *PRB* **1999**, *59*, 1758-1775. <http://doi.org/10.1103/PhysRevB.59.1758>

[11]	K. B. John P. Perdew, Matthias Ernzerhof, *Phys. Rev. Lett.* **1996**, *77*, 3865-3868. <http://doi.org/10.1103/physrevlett.77.3865>

[12]	J. F. G. Kresse, *PRB* **1996**, *54*, 11169. <http://doi.org/10.1103/PhysRevB.54.11169>

[13]	H. J. Monkhorst, J. D. Pack, *PRB* **1976**, *13*, 5188-5192. <http://doi.org/10.1103/PhysRevB.13.5188>

[14]	V. I. Anisimov, J. Zaanen, O. K. Andersen, *PRB* **1991**, *44*, 943-954. <http://doi.org/10.1103/PhysRevB.44.943>

[15]	W. Dollase, *J. Appl. Crystallogr.* **1986**, *19*, 267-272. <http://doi.org/doi:10.1107/S0021889886089458>

**Author Contributions**

Y.H., B.Y. and Q.W. contributed to the design of the research and performed experimental data analysis. Y.H. performed the main part of the synthesis, collected and analyzed the data, and drafted the manuscript. B.Y., St. S., P.N., helped to conduct the sXAS experiments and analyzed the data. W.L. and P.Z. conducted the ARC experiments. C.H. and Chao. L. conducted the NMR experiments. T.Z and Y.X. conducted the NPD experiments. J.X., Y.T. conducted the OEMS. K.Z. prepared the RIXS/sXRD samples and G. Z. collected the spectra and helped with the data analysis. Chunpu. L. helped with the TOF-SIMS data analysis. H.C., T.Q., J.Y., C.Q. provided assistance for this work. M. Y., J-W. W., F. N., Y.Q., S-G.S. supervised the work. All authors have given approval to the final version of the manuscript.
